# Combinatorial optimization enhanced by shallow quantum circuits with 104 superconducting qubits

**DOI:** 10.1093/nsr/nwag124

**Published:** 2026-03-02

**Authors:** Xuhao Zhu, Zuoheng Zou, Feitong Jin, Pavel Mosharev, Maolin Luo, Yaozu Wu, Jiachen Chen, Chuanyu Zhang, Yu Gao, Ning Wang, Yiren Zou, Aosai Zhang, Fanhao Shen, Zehang Bao, Zitian Zhu, Jiarun Zhong, Zhengyi Cui, Yihang Han, Yiyang He, Han Wang, Jia-Nan Yang, Yanzhe Wang, Jiayuan Shen, Gongyu Liu, Zixuan Song, Jinfeng Deng, Hang Dong, Pengfei Zhang, Chao Song, Zhen Wang, Hekang Li, Qiujiang Guo, Man-Hong Yung, Haohua Wang

**Affiliations:** School of Physics, ZJU-Hangzhou Global Scientific and Technological Innovation Center, and Zhejiang Key Laboratory of Micro-nano Quantum Chips and Quantum Control, Zhejiang University, Hangzhou 310027, China; Huawei Technologies Co., Ltd, Shenzhen 518000, China; School of Physics, ZJU-Hangzhou Global Scientific and Technological Innovation Center, and Zhejiang Key Laboratory of Micro-nano Quantum Chips and Quantum Control, Zhejiang University, Hangzhou 310027, China; Huawei Technologies Co., Ltd, Shenzhen 518000, China; Huawei Technologies Co., Ltd, Shenzhen 518000, China; School of Physics, ZJU-Hangzhou Global Scientific and Technological Innovation Center, and Zhejiang Key Laboratory of Micro-nano Quantum Chips and Quantum Control, Zhejiang University, Hangzhou 310027, China; School of Physics, ZJU-Hangzhou Global Scientific and Technological Innovation Center, and Zhejiang Key Laboratory of Micro-nano Quantum Chips and Quantum Control, Zhejiang University, Hangzhou 310027, China; School of Physics, ZJU-Hangzhou Global Scientific and Technological Innovation Center, and Zhejiang Key Laboratory of Micro-nano Quantum Chips and Quantum Control, Zhejiang University, Hangzhou 310027, China; School of Physics, ZJU-Hangzhou Global Scientific and Technological Innovation Center, and Zhejiang Key Laboratory of Micro-nano Quantum Chips and Quantum Control, Zhejiang University, Hangzhou 310027, China; School of Physics, ZJU-Hangzhou Global Scientific and Technological Innovation Center, and Zhejiang Key Laboratory of Micro-nano Quantum Chips and Quantum Control, Zhejiang University, Hangzhou 310027, China; School of Physics, ZJU-Hangzhou Global Scientific and Technological Innovation Center, and Zhejiang Key Laboratory of Micro-nano Quantum Chips and Quantum Control, Zhejiang University, Hangzhou 310027, China; School of Physics, ZJU-Hangzhou Global Scientific and Technological Innovation Center, and Zhejiang Key Laboratory of Micro-nano Quantum Chips and Quantum Control, Zhejiang University, Hangzhou 310027, China; School of Physics, ZJU-Hangzhou Global Scientific and Technological Innovation Center, and Zhejiang Key Laboratory of Micro-nano Quantum Chips and Quantum Control, Zhejiang University, Hangzhou 310027, China; School of Physics, ZJU-Hangzhou Global Scientific and Technological Innovation Center, and Zhejiang Key Laboratory of Micro-nano Quantum Chips and Quantum Control, Zhejiang University, Hangzhou 310027, China; School of Physics, ZJU-Hangzhou Global Scientific and Technological Innovation Center, and Zhejiang Key Laboratory of Micro-nano Quantum Chips and Quantum Control, Zhejiang University, Hangzhou 310027, China; School of Physics, ZJU-Hangzhou Global Scientific and Technological Innovation Center, and Zhejiang Key Laboratory of Micro-nano Quantum Chips and Quantum Control, Zhejiang University, Hangzhou 310027, China; School of Physics, ZJU-Hangzhou Global Scientific and Technological Innovation Center, and Zhejiang Key Laboratory of Micro-nano Quantum Chips and Quantum Control, Zhejiang University, Hangzhou 310027, China; School of Physics, ZJU-Hangzhou Global Scientific and Technological Innovation Center, and Zhejiang Key Laboratory of Micro-nano Quantum Chips and Quantum Control, Zhejiang University, Hangzhou 310027, China; School of Physics, ZJU-Hangzhou Global Scientific and Technological Innovation Center, and Zhejiang Key Laboratory of Micro-nano Quantum Chips and Quantum Control, Zhejiang University, Hangzhou 310027, China; School of Physics, ZJU-Hangzhou Global Scientific and Technological Innovation Center, and Zhejiang Key Laboratory of Micro-nano Quantum Chips and Quantum Control, Zhejiang University, Hangzhou 310027, China; School of Physics, ZJU-Hangzhou Global Scientific and Technological Innovation Center, and Zhejiang Key Laboratory of Micro-nano Quantum Chips and Quantum Control, Zhejiang University, Hangzhou 310027, China; School of Physics, ZJU-Hangzhou Global Scientific and Technological Innovation Center, and Zhejiang Key Laboratory of Micro-nano Quantum Chips and Quantum Control, Zhejiang University, Hangzhou 310027, China; School of Physics, ZJU-Hangzhou Global Scientific and Technological Innovation Center, and Zhejiang Key Laboratory of Micro-nano Quantum Chips and Quantum Control, Zhejiang University, Hangzhou 310027, China; School of Physics, ZJU-Hangzhou Global Scientific and Technological Innovation Center, and Zhejiang Key Laboratory of Micro-nano Quantum Chips and Quantum Control, Zhejiang University, Hangzhou 310027, China; School of Physics, ZJU-Hangzhou Global Scientific and Technological Innovation Center, and Zhejiang Key Laboratory of Micro-nano Quantum Chips and Quantum Control, Zhejiang University, Hangzhou 310027, China; School of Physics, ZJU-Hangzhou Global Scientific and Technological Innovation Center, and Zhejiang Key Laboratory of Micro-nano Quantum Chips and Quantum Control, Zhejiang University, Hangzhou 310027, China; School of Physics, ZJU-Hangzhou Global Scientific and Technological Innovation Center, and Zhejiang Key Laboratory of Micro-nano Quantum Chips and Quantum Control, Zhejiang University, Hangzhou 310027, China; School of Physics, ZJU-Hangzhou Global Scientific and Technological Innovation Center, and Zhejiang Key Laboratory of Micro-nano Quantum Chips and Quantum Control, Zhejiang University, Hangzhou 310027, China; School of Physics, ZJU-Hangzhou Global Scientific and Technological Innovation Center, and Zhejiang Key Laboratory of Micro-nano Quantum Chips and Quantum Control, Zhejiang University, Hangzhou 310027, China; Hefei National Laboratory, Hefei 230088, China; School of Physics, ZJU-Hangzhou Global Scientific and Technological Innovation Center, and Zhejiang Key Laboratory of Micro-nano Quantum Chips and Quantum Control, Zhejiang University, Hangzhou 310027, China; Hefei National Laboratory, Hefei 230088, China; School of Physics, ZJU-Hangzhou Global Scientific and Technological Innovation Center, and Zhejiang Key Laboratory of Micro-nano Quantum Chips and Quantum Control, Zhejiang University, Hangzhou 310027, China; Hefei National Laboratory, Hefei 230088, China; School of Physics, ZJU-Hangzhou Global Scientific and Technological Innovation Center, and Zhejiang Key Laboratory of Micro-nano Quantum Chips and Quantum Control, Zhejiang University, Hangzhou 310027, China; Hefei National Laboratory, Hefei 230088, China; Huawei Technologies Co., Ltd, Shenzhen 518000, China; School of Physics, ZJU-Hangzhou Global Scientific and Technological Innovation Center, and Zhejiang Key Laboratory of Micro-nano Quantum Chips and Quantum Control, Zhejiang University, Hangzhou 310027, China; Hefei National Laboratory, Hefei 230088, China

**Keywords:** combinatorial optimization, Ising model, quantum sampling, superconducting qubit, hybrid quantum-classical algorithm, quantum speedup

## Abstract

A pivotal task for quantum computing is to speed up the solution of problems that are both classically intractable and practically valuable. Among these, combinatorial optimization problems have attracted tremendous attention due to their broad applicability and natural fitness to Ising Hamiltonians. Here we propose a quantum-sampling strategy, on the basis of which we design an algorithm to accelerate the solution of the ground states of the Ising model, a class of Nondeterministic Polynomial time (NP)-hard problems in combinatorial optimization. The algorithm employs a shallow-circuit quantum-sampling subroutine to navigate the energy landscape. Using up to 104 superconducting qubits, we experimentally demonstrate that this algorithm outputs favorable solutions compared with even a highly optimized classical simulated-annealing algorithm, and we illustrate the path toward quantum speedup based on the time-to-solution metric relative to simulated annealing under serial execution. Our results indicate a promising alternative to classical heuristics for combinatorial optimization, for which quantum advantage may become possible on near-term superconducting quantum processors.

## Teaser

Researchers have demonstrated Qjump, a hybrid quantum-classical algorithm on 104 superconducting qubits, which aims at solving combinatorial optimization problems with the potential to outperform a highly optimized simulated annealing algorithm.

Combinatorial optimization is prevalent in science and engineering. Many such problems can be effectively mapped to solving for the ground states of the Ising model, which generally belong to the complexity class Nondeterministic Polynomial time (NP)-hard [1,2]. The difficulty originates from the exponentially large solution space and the non-convexity of the corresponding energy landscape, which frequently traps optimizers in local minima. Classical escape strategies typically involve flipping a subset of spins in the current solution [3–6]. However, these perturbations face a fundamental trade-off: if too weak, they fail to escape the local energy basin; if too strong, they disrupt the algorithm’s memory, akin to a random restart. Despite extensive research, developing an efficient solver for the Ising model remains an ongoing challenge.

Quantum computing, with its exploding computational space offers a natural platform for encoding and solving the Ising problems. Capable of querying the energy function in quantum superposition, a quantum computer can propose new solutions based on the entire energy landscape, thereby facilitating efficient escape from energy basins through quantum interference established in Hilbert space while maintaining the algorithm’s memory. However, experimental realization of quantum speedup for combinatorial optimization is impeded by noise inherent in current quantum processors [7–12]. Since a universal fault-tolerant quantum computer remains distant, considerable effort has been devoted to developing quantum algorithms that are resilient to noise. A paradigmatic example is the quantum approximate optimization algorithm (QAOA) [13,14], which implements a depth-*Q* quantum circuit containing $2Q$ variational parameters optimized through classical feedback loops to obtain approximate solutions. Despite its promise, this approach faces practical challenges, particularly the trainability problem [15–17] and the limited solution quality imposed by hardware-constrained circuit depth [7,18,19].

Here, we develop a quantum-sampling strategy and experimentally demonstrate a hybrid quantum-classical algorithm, termed quantum enhanced jumping (Qjump), to address the aforementioned challenges. In particular, we propose a new truncated parameter-setting scheme characterized by shallow quantum circuits for Qjump sampling to guide the search procedure, which stimulates transitions only between promising energy basins while leaving other computational tasks to a classical computer [20–22]. We evaluate its performance on a superconducting quantum processor by solving Ising problems of up to 104 qubits, with experimental evidence of enhanced solution quality compared with algorithms such as QAOA and simulated annealing (SA). We further envision a quantum hardware with specifications to realize quantum speedup against SA running on a single-core CPU, which is feasible on the basis of available superconducting-qubit technologies. Our results highlight the potential applicability of noisy intermediate-scale quantum (NISQ) processors, combined with hybrid quantum-classical algorithms, to solving real-world problems.

We target the problem of finding the ground state of a general Ising model, a canonical NP-hard problem with broad practical applications [1,2], which can be formulated by an *N*-qubit Ising Hamiltonian


(1) \begin{eqnarray*}
H_{\rm {Ising}} = -\sum _{\lbrace j,k\rbrace \in N} J_{jk}\sigma _j^z \sigma _k^z - \sum _{j=1}^N h_j \sigma _j^z,
\end{eqnarray*}

where $J_{jk}$ denotes the coupling strength between qubit pair {*j, k*}, $\sigma _j^z$ is the Pauli-*Z* operator and $h_j$ characterizes the local magnetic field. As this Hamiltonian typically corresponds to a rugged energy landscape spanned by spin configurations denoted by the bitstring $\boldsymbol {s}$ (e.g. $0101\cdots 0110$), the optimization goal is to find the $\boldsymbol {s}$ that approaches the global minimum energy $E(\boldsymbol {s})=\langle \boldsymbol {s}|H_{\rm Ising}|\boldsymbol {s}\rangle$. In this work we consider qubits arranged on a square lattice with nearest-neighbor couplings only, matching the two-dimensional connectivity of our quantum processor; the parameters $J_{jk}$ and $h_j$ take arbitrary real values.

We focus on challenging problem instances, as determined by the sets of $J_{jk}$ and $h_j$ values in Equation (1), for which classical algorithms such as SA struggle but our quantum algorithm may offer improvement. To identify such instances, 4000 random instances are first generated, each with $J_{jk}$ and $h_j$ drawn independently from zero-mean normal distributions with variances 4 and 1, respectively, and then filtered using an Ising-specialized SA solver (SimAn [23]) running on a single-core CPU (2.3 GHz). Twenty problem instances with the highest times to solution (TTS), ranging from 0.02 to 0.4 s, are selected for the benchmark purpose. We adopt the standard metric ${\rm TTS} = t_{\rm r}\log (1-0.99)/ \log (1-p_{{\rm s}})$, which specifies the time required to reach the global minimum with $99\%$ probability, where $t_{\rm r}$ ($p_{{\rm s}}$) denotes the time (success probability of sampling the global minimum) per run of the algorithm.

It should be noted that planar Ising models of this kind can be solved efficiently by certain classical algorithms that directly exploit planar connectivity, such as the bucket-elimination algorithm [24]. However, the performance of these algorithms typically degrades rapidly when random and/or dense beyond-nearest-neighbor connections are introduced. By contrast, general-purpose heuristics such as SA and Qjump operate solely on the energy landscape and do not exploit information about problem connectivity, and may therefore remain effective in more complicated cases. This consideration justifies our choice of SA as the classical benchmark.

**Figure 1.**
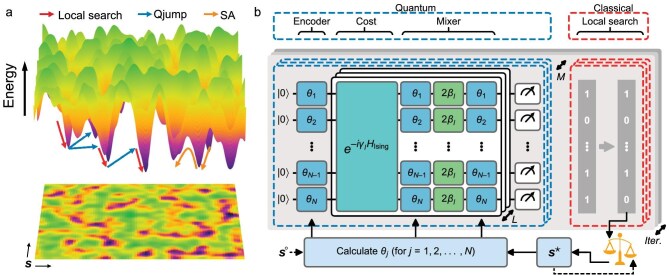
Ising-model energy landscape and schematic of the Qjump algorithm. (a) Illustration of a rugged energy landscape of the Ising model defined in Equation (1), with the $XY$ coordinates labelled by spin configurations $\boldsymbol {s}$. Typical optimization trajectories for both the Qjump and SA algorithms are indicated by colored arrows. (b) Schematic of the Qjump algorithm, featuring the quantum-classical workflow. Within the quantum component, the single-qubit gates represented by colored squares—blue (green) in layer *l*—correspond to Bloch-sphere rotations about the *Y* axis (*Z* axis), i.e., ${Y}(\theta _j)= e^{-i{\theta _j}\sigma ^y/{2}}$  $({Z}(2\beta _l)= e^{-i\beta _l \sigma ^z})$. The cost operator $H_{\rm {Ising}}$ is defined in Equation (1).

For the rugged energy landscape shown in Fig. 1a, our proposed Qjump algorithm leverages a quantum-sampling circuit that, given an initial bitstring $\boldsymbol {s}^\circ$ and repeated for multiple (e.g. *M*) rounds, outputs *M* candidate bitstrings representing jumps to nearby low-energy basins. A classical local search is then performed on all *M* bitstrings to return an optimal solution $\boldsymbol {s}^\star$ corresponding to the lowest explored energy. The quantum-sampling circuit consists of an encoder layer to generate superpositions, *L* layers of cost and mixer operators to enable quantum jumps, and a layer of projective measurements to sample one of the jumps by returning a candidate bitstring for the *N* qubits. Inspired by QAOA, the cost operator encodes a problem instance of $H_{\rm {Ising}}$ (Equation (1)), and the mixer operator implements a mixer Hamiltonian that does not commute with $H_{\rm {Ising}}$. As shown in the digital-circuit schematic (Fig. 1b), the parameters requiring feedback are the rotation angles $\theta _j = 2\arcsin ( \sqrt{0.5 + (s_j - 0.5)\alpha } )$, with mixing coefficient $\alpha \in [0,1]$, where $s_j$ denotes the *j*th bit in either a given initial input $\boldsymbol {s}^\circ$ for a warm start [25] or the best prior solution $\boldsymbol {s}^\star$ if the procedure is iterated. This configuration interpolates between a uniform superposition ($\alpha =0$) and a deterministic bias toward the input bitstring ($\alpha =1$); in practice, $\alpha$ is typically set to an intermediate value related to the bit-flip ratio of the output $\boldsymbol {s}^\star$ relative to the input bitstring. Variational parameters used in the cost and mixer operators, namely $\gamma _l$ and $\beta _l$ for layer *l*, are predefined using the parameter-setting heuristic of a depth-*Q* QAOA ansatz [26–28] (see Section S2C). The entire Qjump algorithm typically cycles between the quantum and classical circuits shown in Fig. 1b multiple iterations ($\alpha \equiv 0$ in the first iteration) until a desired solution is obtained.

A QAOA circuit with large *Q* can approximate adiabatic quantum annealing, leading to a high probability of finding the global energy minimum. Our analysis of QAOA circuit dynamics (Section S2) indicates that the first few layers primarily facilitate broad-range explorations, whereas the later layers function analogously to classical local search. We therefore design the Qjump sampling circuit by retaining the first *L* layers from a depth-*Q* parameter-set QAOA circuit, referred to as an [$L,Q$]-sampler. In contrast to QAOA, the Qjump framework eliminates the need for parameter optimization by sampling from circuits with fixed transfer parameters determined according to [26–28], and strategically replaces quantum circuit segments with classical local-search routines.

**Figure 2.**
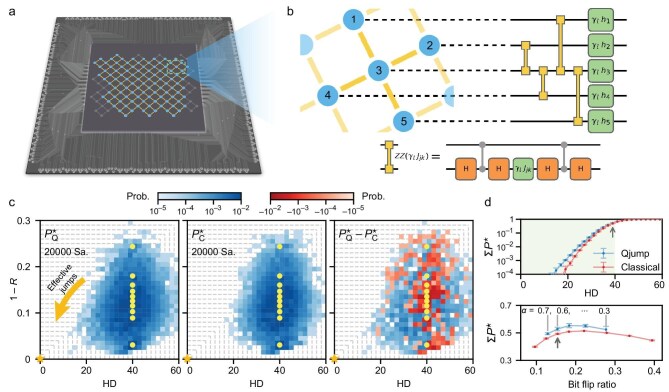
The 104-qubit superconducting quantum processor and performance of the quantum sampler. (a) Illustration of the superconducting quantum processor, in which the 104 qubits (blue dots) and connecting couplers (yellow lines) used in the experiment are highlighted. (b) Circuit schematic for implementing the cost operator $e^{-i\gamma _l H_{{\rm Ising}}}$. Here ${\rm ZZ}(\gamma _l J_{jk}) = e^{-i{\gamma _l J_{jk}} \sigma ^z_j \sigma ^z_k/{2}}$, which is compiled into a series of Hadamard gates (orange squares), virtual *Z*-axis rotations with the indicated angles (green squares) and CZ gates (gray dots connected by lines). (c) Sampling probability $P^{\star }_{{\rm Q}}$ (left) as a function of energy $1-R$ and Hamming distance (HD) for Qjump with quantum sampling, compared with classical random sampling $P^{\star }_{{\rm C}}$ (middle). The probabilities are obtained by counting bitstrings within the colored unit boxes of size 0.01 in $1-R$ and 2 in HD, while the total number of bitstrings for normalization is indicated. The data correspond to problem instance $\#1$ (see Section S3A), with 10 initial $\boldsymbol {s}^{\circ }$, all at ${\rm HD} = 40$ (marked by yellow circles). Also shown is the difference between $P^{\star }_{{\rm Q}}$ and $P^{\star }_{{\rm C}}$ (right). Since *effective jumps* (illustrated by the thick golden arrow in the $P^{\star }_{{\rm Q}}$ panel) refer to sampled bitstrings that are closer to the global minimum than $\boldsymbol {s}^{\circ }$ in terms of both HD and energy, we identify them by outlining a series of square regions anchored at the origin with gray dashed lines, indexed by Hamming distance to the global minimum. We note that the aspect ratio of the square regions can be chosen quite flexibly, yielding similar conclusions in the performance benchmark. (d) Top: sum of probabilities within the square regions outlined in (c), $\sum P^{\star }$, as a function of the region index (HD). Bottom: $\sum P^{\star }$ within the square region indexed by ${\rm HD} = 40$, corresponding to all *effective jumps* in a single step, as a function of the bit-flip ratio for Qjump, with the corresponding $\alpha$ values indicated (blue). Classical random-sampling results are shown for comparison (red). Error bars represent the standard deviations over five repeated experiments.

We experimentally investigate the Qjump algorithm on a superconducting quantum processor comprising 104 frequency-tunable transmon qubits arranged on a two-dimensional square lattice (Fig. 2a). Controlled $\pi$-phase (CZ) gates can be implemented between any two neighboring qubits, with a median gate fidelity of ${\sim }99.5\%$. All 104 qubits can be simultaneously manipulated by arbitrary rotation gates and jointly measured in the computational basis, as required in the Qjump circuit (Fig. 1b), with median single-qubit gate and measurement fidelities of ${\sim }99.95\%$ and ${> }99\%$, respectively. The cost operator $e^{-i\gamma _l H_{\rm {Ising}}}$ in Qjump is compiled into a digital sequence comprising eight layers of CZ gates, with a layer of virtual *Z* gates (implemented by adding phase shifts to subsequent microwave rotations) and single-qubit rotation gates inserted between successive CZ layers. Gate twirling is used to the CZ gates to mitigate noise [9] (See Section S4 for detailed device and circuit information).

We begin by examining the performance of the quantum sampler for a given problem instance, denoted instance #1 (see Section S3A), and initial guesses $\boldsymbol {s}^\circ$, focusing on its sampling probability $P^\star$ toward the global minimum in single-step jumps. Here, $P^\star$ denotes the probability that a sampled bitstring appears within a unit area in a two-dimensional plane spanned by energy and Hamming distance (HD). The energy scale is defined as $1-R$, with approximation ratio $R= E_{{\rm exp}} / E_{{\rm g}}$, where $E_{{\rm exp}}$ is the experimentally obtained Ising energy of the sampled bitstring and $E_{{\rm g}} < 0$ is the ground-state energy; HD is defined as the number of flipped qubit sites relative to the global optimal bitstring. We enumerate 10 initial $\boldsymbol {s^\circ }$ separated from the global minimum by the same HD (e.g. 40), and experimentally run the Qjump algorithm with $M=2000$ for one iteration, yielding 2000 bitstrings for each $\boldsymbol {s^\circ }$ and a total of 20000 bitstrings to estimate $P^\star$. Defining the unit area as a box of size 0.01 in $1-R$ and 2 in HD, a scatter plot of $P^\star$ on the energy-versus-HD plane is obtained in Fig. 2c (left). Comparing the $20\, 000$ bitstrings with $\boldsymbol {s^\circ }$, we find that the bit-flip ratio approximately follows a normal distribution, from which the mean bit-flip ratio of the quantum sampler is extracted.

For the predefined parameters required to run Qjump, we adopt the variational parameter values $\gamma _l$ and $\beta _l$ from a depth-*Q* QAOA ansatz, and examine different *Q* values up to 20 [26–28]. We focus on the shallow-circuit regime by considering layer number $L\in [1,3]$ with $\alpha \in [0.3,0.7]$ for the circuit diagram in Fig. 1b. We find that Qjump is quite robust with respect to different choices of predefined parameters, all demonstrating promising capability in reaching the vicinity of the global minimum across various problem instances. In the following benchmarks, we adopt the $[{L}=2, {Q}=20]$-sampler, with circuit depth 33, comprising 1590 single-qubit and 728 two-qubit gates for 104 qubits, and $\alpha =0.6$, unless otherwise specified, for illustrative purpose. We emphasize that the rigorous parameter optimization for Qjump with more than 100 qubits is extremely resource-consuming and is not attempted here.

To demonstrate the effectiveness of quantum sampling, we replace the quantum component in Fig. 1b with classical random sampling, which stochastically flips certain qubit sites in a given $\boldsymbol {s}^\circ$ with a flipping ratio equal to the mean value obtained for quantum sampling. For problem instance $\#1$, the scatter plot of $P^\star$ under random sampling with similar conditions is shown in Fig. 2c (middle), in contrast to the quantum-sampler result, highlighting that Qjump more favorably explores regions close to the global minimum at the origin [Fig. 2c (right)]. Since *effective jumps* refer to sampled bitstrings closer to the global minimum in both HD and energy, we further quantify the sampling difference by defining square regions anchored at the global minimum and progressively shrinking their sizes (guided by a series of gray dashed boundaries and indexed by HD from the origin). The cumulative probability $\sum P^\star$ within these square regions is shown in Fig. 2d (top), where a larger $\sum P^\star$ at smaller HD indicates better performance. As sampled bitstrings with $\rm {HD}< 40$ constitute effective jumps, Fig. 2d (bottom) presents the effective-jump probabilities at different $\alpha$ values, compared with classical sampling at similar bit-flip ratios. We observe that the experimental quantum sampler outperforms its classical counterpart, even in the presence of experimental imperfections such as non-ideal quantum gates and measurements. This behaviour is the observed for the majority of the problem instances.

We now quantify the solution quality of the Qjump algorithm within a quantum-classical hybrid workflow, in comparison with the popular QAOA and SA algorithms, based on 20 problem instances of the 104-qubit Ising model. We examine the likelihood that the solutions (bitstrings) approach the global minimum, under the prerequisite that our comparison must take into account the different algorithmic run times. Returning one optimal bitstring per run, the algorithms can yield substantially different numbers of bitstrings within the same period due to their distinct algorithmic structures and parameter settings. For a fair comparison, rather than plotting sample probabilities as in Fig. 2, here and below we analyse the distribution of sample occurrences—namely, the counts of bitstrings within unit boxes, without normalization—on the energy-HD plane. Larger counts near the global minimum indicate better solution quality.

Our experimental setup cannot execute the algorithm sufficiently rapidly to generate a large number of bitstrings within a short period to win the competition (see Section S3C for details). Instead, we consider the feasibility of constructing state-of-the-art quantum hardware, based on available superconducting-qubit technologies, and assess whether such hardware could demonstrate quantum speedup by running Qjump in comparison with SA on a single-core CPU. To this end, we assume that the envisaged quantum hardware, analogous to the requirements for quantum error correction, can execute a sequence of operations—including qubit reset, gate operations, measurement, classical local search and comparison and feedback control for subsequent gate settings—fully controlled by a field-programmable gate array (FPGA) with high programming flexibility and without reliance on an external classical computer. According to reported speeds of quantum operations and measurements, the quantum sampler could reach a sampling frequency of 0.4 MHz [29–31], while the FPGA can process classical information at a rate comparable to that of the CPU running SA (see Section S3C for the Qjump workflow).

Under these assumptions, and based on the actual runtimes of classical operations, we first determine the number of bitstrings that each algorithm could produce within a fixed period of 40 ms. We then repeatedly run Qjump with 12 iterations and QAOA with $Q=6$ on our superconducting processor, and SA with 700 sweeps on a single-core CPU, to generate these predetermined numbers of bitstrings for analysis. Classical local search is appended to QAOA, which is necessary for the algorithm to truly locate the global minimum. QAOA with $Q=6$ has a circuit depth of 97 layers, comprising approximately 4500 single-qubit gates and 2100 CZ gates. The optimal cycling parameter for each algorithm, i.e. the number of iterations (Qjump), the number of sweeps (SA) or the circuit depth (QAOA), is selected such that the TTS approaches its best value (see below).

**Figure 3.**
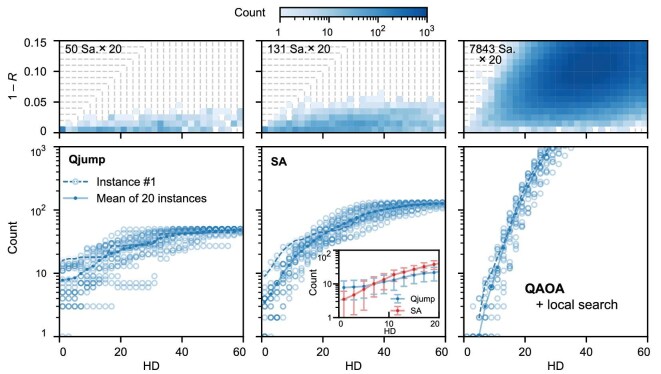
Performance of Qjump in comparison with SA and QAOA. Solution distributions of Qjump, SA and QAOA, summarized over 20 instances. Here, Qjump uses $L=2,Q=20,\alpha =0.6$; SA uses 700 sweeps (see Section S3B for parameter details); QAOA uses $Q=6$ without iterative parameter updates. Top row: distribution of solution counts over 20 problem instances for each algorithm. Bottom row: solution counts for individual instances and their means as functions of HD toward the global minimum, processed in a manner similar to that in Fig. 2d. Based on the computational speeds of the envisioned quantum hardware and the classical CPU (See Section S3C for the estimated time per run: Qjump $\sim 0.78$ ms; SA $\sim 0.30$ ms; QAOA $\sim 5.1\mu$s), we first determine the numbers of bitstrings that can be generated by repeated runs of these algorithms within a fixed period of 40 ms (as indicated in the figure panels for the 20 instances). We then run Qjump and QAOA on our superconducting processor to generate the predetermined numbers of bitstrings for analysis, which requires substantially longer times in our experimental setup. Mean values extracted from Qjump and SA are shown in the panel inset, with error bars representing the standard deviations over the 20 instances. On average, Qjump samples the global minimum 2.23 times more frequently than SA for the 104-qubit Hamiltonians.

The resulting distributions of sample occurrences on the energy-HD plane, averaged over the 20 problem instances by Qjump, SA and QAOA are displayed side by side in the top row of Fig. 3. Although Qjump yields fewer bitstrings (50 per instance) within 40 ms, it appears to explore close to the global minimum with more weights. To further quantify this difference, we plot, in the bottom row of Fig. 3, the sample occurrences over the 20 instances as functions of square-region index HD toward the origin. As HD goes to zero, the averaged sample occurrence for Qjump is highest, demonstrating its superior performance in reaching the global minimum.

**Figure 4.**
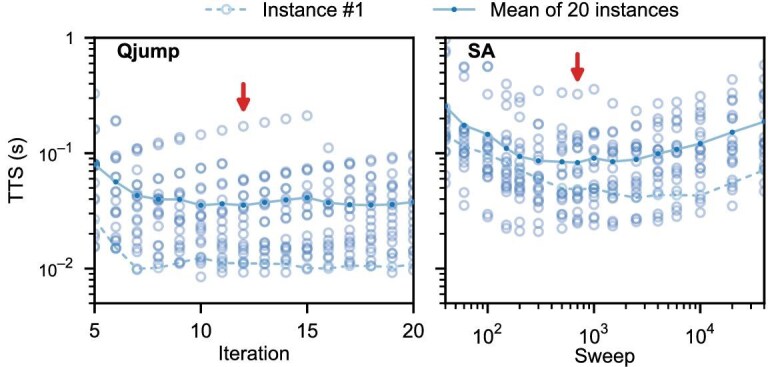
Time-to-solution (TTS) estimation for Qjump and SA. Each open circle represents the TTS value for a given problem instance obtained by running the Qjump (SA) algorithm with the specified iterations (sweeps). Dots connected by lines denote the mean over the 20 problem instances, and the arrow indicates the optimal iteration/sweep number adopted for the algorithmic benchmark in Fig. 3. According to the minimal TTS values, Qjump on the envisioned quantum hardware outperforms SA on a single-core CPU by an average factor of 2.34 for the 104-qubit Ising model (see Section S4D for cases with fewer qubits). TTS values for instance $\#1$ are highlighted by dashed lines. QAOA, even when appended with classical local search, is largely ineffective in reaching the global minimum; its TTS values, estimated to exceed 4 s, are therefore not shown.

Now we discuss the speedup potential of Qjump, based on the aforementioned assumptions regarding the envisioned quantum hardware and the known runtimes of classical operations. Figure 4 presents the TTS estimates for Qjump and SA (data for QAOA are not shown), from which we select the optimal cycling parameter for each algorithm. More importantly, given currently available superconducting-qubit technologies, Qjump running on the envisioned quantum hardware could outperform SA on a single-core CPU by a factor of 2.34 in terms of the TTS metric, consistent with the results in Fig. 3. Of course, the performance of classical heuristics can be substantially enhanced through parallel computing techniques, for example using GPUs, which are not considered here.

Nevertheless, the potential advantage over a single-core CPU remains encouraging and raises several open and interesting questions, particularly if the benchmark is extended to larger scales, higher connectivities and more diverse coupler-weight distributions. On the one hand, the scaling performance of approximation optimization heuristics is known to be unstable at small system sizes [32]. On the other hand, due to the limited size of the present experiment, the time complexity of Qjump is not yet understood, and the eventual answer will depend critically on progress in scaling up experimental demonstrations. Meanwhile, the influence of gate and measurement infidelities on the shallow-circuit Qjump sampler remains unclear. It will be interesting to explore how to rigorously optimize the quantum sampler with respect to both parameter settings and circuit structure.

As we demonstrated in this work, the hybrid workflow of Qjump, which judiciously combines efficient aspects of classical and quantum subsystems, shows promise for achieving an advantage over previously known algorithms. In particular, the pre-trained parameter setting scheme avoids variational updates and thus mitigates the risk of encountering barren plateaus. Circuit truncation, together with the use of steepest-descent local search, enhances the algorithm’s robustness against noise by reducing circuit depth and offloading the exploration of subtle landscape features to the classical subsystem. At the same time, we have shown that sampling from shallow quantum circuits, combined with the novel use of a truncated parameter-setting strategy, can serve as an efficient tool for broad landscape exploration and escape from local minima, which is the most challenging part of classical optimization. This result relates to previous theoretical works on quantum advantage with shallow quantum circuits [33–35], although we do not claim quantum advantage here in the strong sense.

Here, we have considered only Ising problems with nearest-neighbor connections, which may be handled by structure-specific solvers such as the bucket-elimination algorithm. More challenging Ising instances with higher connectivity could be addressed either by directly engineering on-chip long-range couplings [36] or by implementing SWAP networks on a planar topology [37], the latter requiring a number of SWAP gates that scales polynomially with the qubit number. Also, practical optimization problems typically involve thousands to millions of variables, far exceeding the 104 qubits demonstrated in our experiment. It would therefore be interesting to implement Qjump on processors with thousands of qubits or more, together with further improved qubit metrics, which may ultimately enable quantum advantage on NISQ devices. Nevertheless, our present 104-qubit demonstration can be regarded as a subsystem that may still provide useful jumping capability [38]. Moreover, since escaping local minima is a common requirement in many optimization algorithms, Qjump could be integrated with traditional tabu search or the Metropolis method, or employed as a heuristic module within exact algorithms, such as branch-and-bound. In practice, different heuristics—whether quantum or classical—can exhibit substantial performance deviations across problem instances, necessitating algorithm selection [39]. From this perspective, Qjump represents a promising alternative in the heuristic ensemble.

## Supplementary Material

nwag124_Supplemental_File
