# Supplementary material for: Combinatorial optimization enhanced by shallow quantum circuits with 104 superconducting qubits
Source: Natl Sci Rev. 2026 Mar 2;13(9):nwag124. doi: 10.1093/nsr/nwag124 (PMC13221960; doi:10.1093/nsr/nwag124)
Supplement: nwag124_Supplemental_File [file nwag124_supplemental_file.pdf]

Supplementary Information for
Combinatorial optimization enhanced by shallow quantum circuits with 104 superconducting qubits

CONTENTS

1. A brief overview of combinatorial optimization algorithms 1

2. Theoretical analysis 2
   A. Principle of QAOA 2
   B. Principle of warm-start QAOA 4
   C. Parameter setting scheme 5

3. Numerical simulations 6
   A. Problem instances 6
   B. Simulated annealing 6
   C. Qjump time estimation 11

4. Experimental details 13
   A. Device infomation 13
   B. Quantum circuit 15
   C. Performance of the quantum sampler with different parameters 16
   D. Additional experimental data for different system sizes 16

References 21

1. A BRIEF OVERVIEW OF COMBINATORIAL OPTIMIZATION ALGORITHMS

Despite the rapid development of hardware platforms, the Noisy Intermediate-Scale Quantum (NISQ) era has yet to produce a truly transformative application. This paper introduces a quantum-classical hybrid optimization algorithm for solving combinatorial optimization problems, providing a promising avenue for achieving quantum enhancement.

Many combinatorial optimization problems, such as the Traveling Salesman Problem and the Knapsack Problem, are NP-hard. These problems are ubiquitous in real-world scenarios, including logistics, scheduling, and resource allocation, and require an exhaustive search through an exponentially large space to find the optimal solution. Traditional algorithms for these problems are broadly categorized into two types: exact methods and heuristic methods. Exact methods, such as integer optimization techniques used by commercial solvers like Gurobi, guarantee the accuracy of the solution but are often computationally expensive. Heuristic methods, inspired by physical phenomena such as simulated annealing (SA) [1], tabu search [2] and genetic algorithms [3] iteratively explore the variable space to minimize the objective function.

In this work, we focus on solving the Ising model, as most combinatorial optimization problems can be mapped to its ground state problem [4]. While classical heuristic algorithms can be accelerated using caching techniques to reduce computation time, their exploratory capabilities remain fundamentally limited. Their search steps are fixed and universal, making them less effective for specific problem structures. In contrast, quantum optimization algorithms, such as the Quantum Approximate Optimization Algorithm (QAOA) [5] and its variants [6], the Quantum Imaginary Time Evolution (QITE) algorithm [7, 8], quantum walks [9], and quantum annealing [10], leverage problem-specific information to perform a global search across the quantum state space. By quantum tunneling and quantum interference, these algorithms have the potential to outperform classical methods, as demonstrated by studies [11–13].

One significant challenge for quantum optimization algorithms is the excessive circuit depth required to achieve a quantum advantage. Even the least resource-intensive QAOA requires a considerable number of qubits and layers (e.g., 12 layers for hundreds of qubits, as reported in Ref. [12]), which exceeds the capabilities of current hardware [14, 15]. To address this, we propose a hybrid quantum-classical algorithm, named quantum enhanced jumping (Qjump). By alternating between classical and quantum processors, the algorithm delegates the exploration of the search space to the quantum processor and the refinement of the solution to the classical processor. This hybrid approach can potentially achieve a quantum speedup even with current NISQ hardware.

2. THEORETICAL ANALYSIS

The excellent performance of the Qjump algorithm stems from the use of shallow-depth Warm-started QAOA[16, 17] circuits with novel truncated parameter setting technique, which can enhance the probability of landing in a better energy basin through constructive interference. This contrasts with the classical heuristics that escape local energy basins through random exploration, leading to an equal probability of landing on both poor and good bitstrings. In the following section, we will explain how the quantum circuit achieves this.

A. Principle of QAOA

The standard QAOA starts from an equal superposition state $|+\rangle^{\otimes N} = \frac{1}{\sqrt{2^N}}\sum_{y=1}^{2^N}|\boldsymbol{s}^y\rangle$, where $\boldsymbol{s}^y$ represents all possible bitstrings in the Hilbert space. The algorithm then executes a quantum circuit consisting of alternating cost and mixer layers. For the simplest case of a single-layer QAOA circuit, with γ and β as the parameters for the cost and mixer layers, respectively, the probability amplitude for obtaining a specific bitstring $\boldsymbol{s}^x$ is given by [18]:

$$F_x = \frac{1}{\sqrt{2^N}}\sum_y \left\langle \boldsymbol{s}^x \left| e^{-i\beta H_{\mathrm{M}}} e^{-i\gamma H_{\mathrm{Ising}}} \right| \boldsymbol{s}^y \right\rangle = \frac{1}{\sqrt{2^N}}\sum_y \left\langle \boldsymbol{s}^x \left| e^{-i\beta H_M} \right| \boldsymbol{s}^y \right\rangle e^{-i\gamma E_y}, \tag{S1}$$

where $H_{\mathrm{M}} = \sum_{j=0}^{N} X_j$ and H_{Ising} are the mixer and Ising problem Hamiltonians, respectively. X_j represents a π rotation around the X-axis of the Bloch sphere for qubit j, and E_y is the Ising energy for $\boldsymbol{s}^y$. After cost layer, energies are imprinted onto the phase of the state. Since phase information cannot be directly measured, the mixer layer is used to amplify the amplitudes of the optimal solutions. The inner product can be expressed as $\left\langle \boldsymbol{s}^x \left| e^{-i\beta H_{\mathrm{M}}} \right| \boldsymbol{s}^y \right\rangle = \cos^{N-d_{xy}}(\beta)(-i\sin(\beta))^{d_{xy}}$, where d_{xy} is the Hamming distance between $\boldsymbol{s}^x$ and $\boldsymbol{s}^y$. Thus, F_x can be rewritten as:

$$F_x = \frac{1}{\sqrt{2^N}}[\cos(\beta)]^N \cdot \sum_y e^{-i(\gamma E_y + \frac{\pi}{2} d_{xy})}[\tan(\beta)]^{d_{xy}}. \tag{S2}$$

The sum in this formula can be visualized as the superposition of vectors on complex plane, each corresponding to a bitstring $\boldsymbol{s}^y$. The phase of each vector is determined by its energy and Hamming distance to $\boldsymbol{s}^x$ via expression $\gamma E_y + \frac{\pi}{2} d_{xy}$, while its length is proportional to $[\tan(\beta)]^{d_{xy}}$.

We demonstrate this visualization using a 10-qubit regular-4 Ising model. Since our primary interest is the probability of finding the global minimum, we choose the global optimal bitstring as $\boldsymbol{s}^x$ and plot the distribution of all bitstrings' energies and their Hamming distances from $\boldsymbol{s}^x$ in Fig. S1**a**. To visualize the superposition, we group the vectors by their Hamming distance, and then sort the vectors by their corresponding energies within each distance group. Sequentially connecting these vectors forms an open-ended, meandering curve. The distance between the start and the open end of the curve corresponds to the probability amplitude of obtaining $\boldsymbol{s}^x$.

As shown in Fig. S1**b** and **c**, we display the results for a $Q = 1$ QAOA circuit (with $\beta \approx 0.67$) and the final layer of a $Q = 3$ QAOA circuit (with $\beta \approx 0.23$). For the $Q = 1$ circuit, bitstrings with Hamming distances ranging from 2 to 8 contribute significantly to the overall path. In contrast, for the final layer of the $Q = 3$ circuit, only bitstrings with $d_{xy} \leq 3$ have a significant contribution. This effect is further illustrated in Fig. S2, where we plot the total contributions from bitstrings at different Hamming distances for these two cases. The comparison of these distributions reveals that as β decreases, the contribution from bitstrings with a small Hamming distance becomes significantly more pronounced, despite the exponential growth in the number of bitstrings at larger distances. Note that all numerical simulations of quantum circuit dynamics and sampling were performed using MindQuantum v0.10 software [19].

It should be noted that while these results are for a single QAOA layer, the same principles apply to deeper circuits. The only difference is that the initial superposition state for each layer changes, while the cost and mixer layers themselves function in the same manner. In a sufficiently deep QAOA circuit, the annealing schedule involves a gradual increase in γ and a progressive decrease in β with increasing depth. This behavior effectively causes the later stages of the QAOA circuit to act essentially similar to a classical local search, as the contributions to F_x are dominated by bitstrings within a very narrow Hamming distance.

To validate the effectiveness of these multi-layer circuits on problems that are challenging for classical local search, we simulate QAOA circuits with varying layers (L) on 100 random instances of the 20-qubit regular-4 Ising model. For this simulation, we consider two different QAOA ansatz: $Q = L$ and $Q = 20$. We define the energy basin of a local minima as the set of all bitstrings that can reach that local minima after employing a steepest descent local search. As shown in Fig. S3, in the shallow circuit region for the $Q = 20$ ansatz, the probability of reaching the exact global minimum is smaller than for the $Q = L$ ansatz. However, the probability of reaching the global optimal energy basin

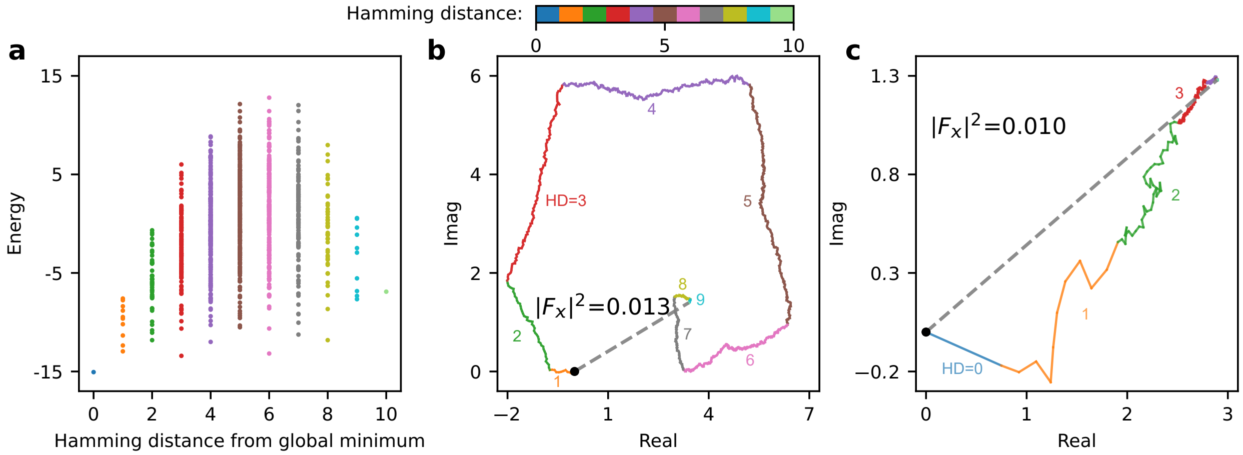

Figure S1. **The energy-Hamming-distance correlation for the QAOA circuit. a.** Energy distribution as a function of Hamming distance from the global minimum. **b.** Complex plane plot of each component of F_x for the $Q = 1$ QAOA circuit ($\beta \approx 0.67$), ordered by Hamming distance. **c.** Complex plane plot of each component of F_x for the final layer of the $Q = 3$ QAOA circuit ($\beta \approx 0.23$), ordered by Hamming distance.

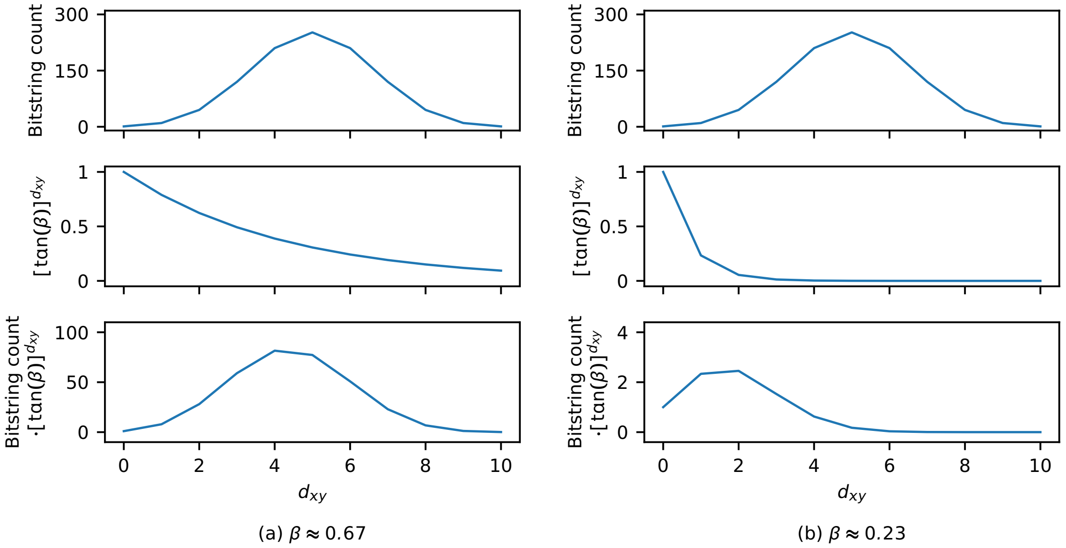

Figure S2. **The contributions from different Hamming distance.** Panels (a) and (b) illustrate the contribution of bitstrings at varying Hamming distances to F_x for $\beta \approx 0.67$ and $\beta \approx 0.23$. Each panel shows, from top to bottom, the count of bitstrings at each Hamming distance, the corresponding $[\tan(\beta)]^{d_{xy}}$ and their product.

is higher. This shows that when used in a hybrid approach with a classical local search with shallow circuits, the $Q = 20$ ansatz produces higher-quality solutions compared to the $Q = L$ ansatz.

For the analysis of large-scale systems, where it is computationally infeasible to calculate the energy of every bitstring or to simulate the quantum circuit, we rely on a mathematical formalization. We adopt the methodology proposed in references [18, 20], which assumes that the joint distribution of energy and Hamming distance follows a bivariate Gaussian distribution. Specifically, we model the energy distribution as $E_y \sim \mathcal{N}(0, \sigma_E^2)$, the Hamming distance distribution as $d_{xy} \sim \mathcal{N}(N/2, N/4)$, assume a non-zero covariance between them, i.e., $\mathrm{Cov}(E_y, d_{xy}) \neq 0$. Furthermore, we consider how the distribution of d_{xy} is affected by the $[\tan(\beta)]^{d_{xy}}$ weighting factor. This weighting term creates a new, biased distribution for the Hamming distance labeled $\tilde{d}_{xy}$. Based on these assumptions, the probability of obtaining a $\boldsymbol{s}^x$ can be approximated as:

$$P(\boldsymbol{s}^x) = |F_x|^2 \sim e^{-\gamma^2\sigma_E^2 - \pi\gamma \mathrm{Cov}(E_y, \tilde{d}_{xy})}, \tag{S3}$$

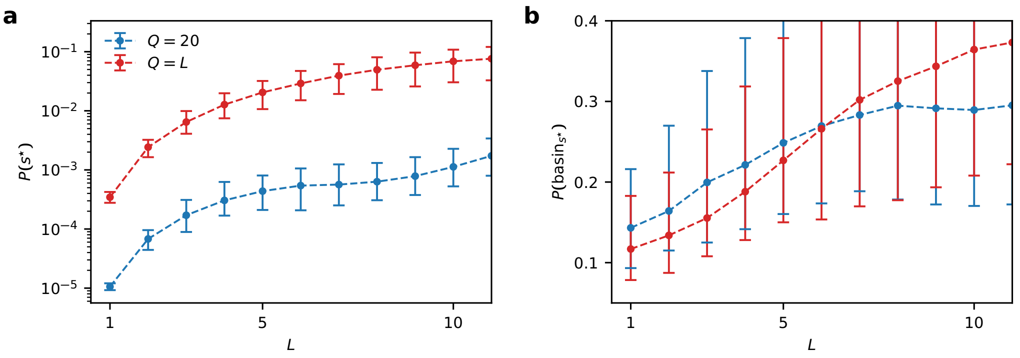

Figure S3. **Numerical simulations of L-layer QAOA circuits with ansatzes of $Q = 20$ and $Q = L$.** The results are averaged over 100 random 20-qubit regular-4 Ising instances with gaussian weights and external fields. The error bars represent the lower and upper quartiles. **a,** The probability of obtaining the exact global optimal solution $s^\star$. **b,** The probability of reaching global optimal basin, where $Q = 20$ have better performance with shallow circuits.

The probability peaks at $\gamma^\star = -\frac{\pi \mathrm{Cov}(E_y, \tilde{d}_{xy})}{2\sigma_E^2}$ with the maximal value of $P^\star(\boldsymbol{s}^x) \sim e^{\pi^2 \mathrm{Cov}(E_y, \tilde{d}_{xy})/4\sigma_E^2}$. For a given σ_E, a stronger energy-Hamming-distance correlation, $\mathrm{Cov}(E_y, \tilde{d}_{xy})$, results in a higher probability of obtaining $\boldsymbol{s}^x$. These results will be used in the next section to analyze the circuit's behavior.

B. Principle of warm-start QAOA

A major limitation of standard QAOA is the restriction on achievable circuit depths due to current hardware constraints. In Qjump, we address this using the technique of Warm-start QAOA (WS-QAOA)[16, 17], which leverages information from a previous iteration to initialize the quantum circuit. This approach focuses the quantum sampling on finding an improved solution within the vicinity of a known solution. Given an initial solution $\boldsymbol{s}^\circ$, the amplitude F_x after a single WS-QAOA layer is given by:

$$F_x = \langle \boldsymbol{s}^x | \boldsymbol{s}^\circ, \theta, \gamma, \beta \rangle = [\cos(\theta/2)]^N \cdot (\cos\beta + i \sin\beta \cos\theta)^N \cdot \sum_y e^{-i\gamma E_y} [\tan(\theta/2)]^{d_{\circ y}} \left(\frac{i \sin\theta \sin\beta}{\cos\beta + i \sin\beta\cos\theta} \right)^{d_{xy}}, \tag{S4}$$

where $\theta = 2 \arcsin\left(\sqrt{0.5 - 0.5\alpha}\right)$, and γ and β are the circuit parameters of the first layer. This formulation introduces an exponential suppression factor, $[\tan(\theta/2)]^{d_{\circ y}}$, which reshapes the initial uniform superposition into a distribution concentrated around $\boldsymbol{s}^\circ$. Specifically, the mixing coefficient $\alpha \in [0, 1]$ controls the degree of the bias towards the initial bitstring $\boldsymbol{s}^\circ$. When $\alpha = 0$, the formula yields $\theta = 2 \arcsin(\sqrt{0.5}) = \pi/2$, and F_x reverts to the standard, unbiased QAOA situation. When $\alpha = 1$, the factor $\tan(\theta/2)$ approaches zero, causing the probability distribution to collapse onto the initial state $\boldsymbol{s}^\circ$.

In comparison with standard QAOA, warm-start QAOA localizes the search space around the initial solution $\boldsymbol{s}^\circ$. When an proper $\boldsymbol{s}^\circ$ is chosen near the global minimum $\boldsymbol{s}^\star$, the basin containing $\boldsymbol{s}^\star$ is favored by the high energy-Hamming-distance correlation, leading to a higher sampling probability. Conversely, bitstrings far from $\boldsymbol{s}^\star$ are negatively affected by this correlation. Therefore, introducing a better initial guess such as the Qjump does for later iterations is helpful.

To demonstrate the distinct behavior of quantum jumping in contrast to its classical counterpart, we performed a numerical simulation on instance #1 from the main text ($N = 104$) with $\alpha = 0.6$ and γ, β from the first layer of a $Q = 20$ QAOA ansatz. We start from $\boldsymbol{s}^\circ$ trapped in local minima (e.g., at a Hamming distance of 10 from $\boldsymbol{s}^\star$). Using a Monte Carlo method and an approximation similar to the one introduced in Section 2A, we sample bitstrings from the conditional distribution $p(\boldsymbol{s}^y | \boldsymbol{s}^x, \boldsymbol{s}^\circ) \sim [\tan(\theta/2)]^{d_{\circ y}} \left|(i \sin\theta \sin\beta)/(\cos\beta + i \sin\beta\cos\theta)\right|^{d_{xy}}$. We define two target states: the global minimum $\boldsymbol{s}^x = \boldsymbol{s}^\star$, and an opposite distant state $\boldsymbol{s}^x = \boldsymbol{s}^{\star\!\!\!\!\!\;\star}$ (e.g. at a Hamming distance 10 from $\boldsymbol{s}^\circ$ and 20 from $\boldsymbol{s}^\star$). Due to the computational intractability of calculating the full covariance over all possible bitstrings, we instead sample 10000 bitstrings for each target state and calculate the local covariance $\rho(d_{xy})$ between the energy E_y and the Hamming distance d_{xy} within a small range (e.g., Hamming distance from $\boldsymbol{s}^y$ within 4). This quantity $\rho(d_{xy})$ correlates with the probability of obtaining the target state $\boldsymbol{s}^x$, with a higher value indicating a higher probability. As shown in Fig. S4, the results for each target state are averaged over 10 different $\boldsymbol{s}^\circ$ and 10 different $\boldsymbol{s}^x$, respectively.

In classical jumping, starting from $\boldsymbol{s}^{\circ}$, the states $\boldsymbol{s}^{\star}$ and $\boldsymbol{s}^{\star}$ have the same Hamming distance and thus an equal probability of being reached. In contrast, quantum jumping simulation shows a very different behavior. As shown in Fig. S4, the energy-Hamming-distance correlations for the target states are clearly different. The global minimum exhibits a much better correlation than the distant ones, which explains why quantum jumping is superior to classical jumping in guiding the search towards the optimal region.

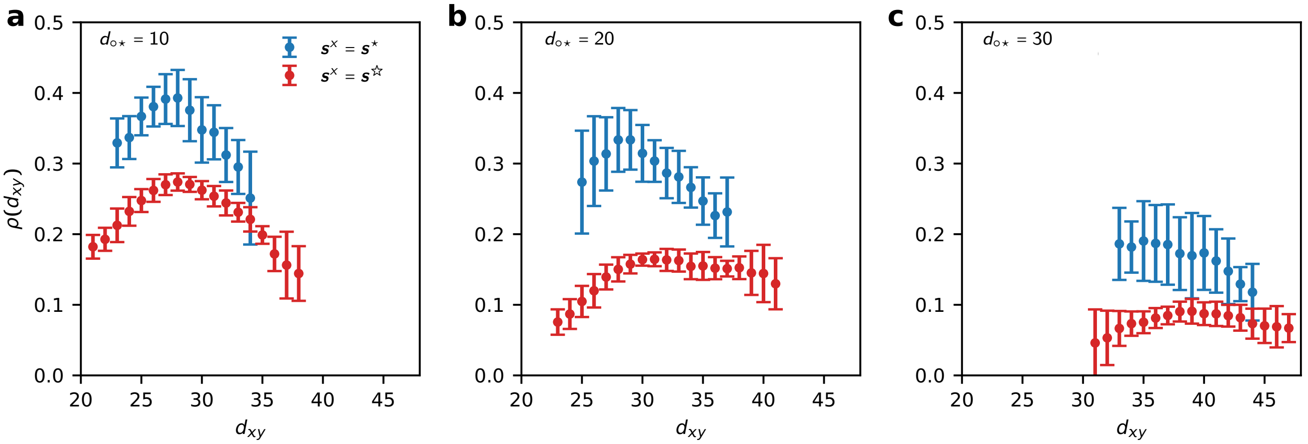

Figure S4. **The Energy-Hamming-distance correlations of bitstrings $\boldsymbol{s}^y$ sampled from the distribution $p(\boldsymbol{s}^y|\boldsymbol{s}^x, \boldsymbol{s}^{\circ})$.** The plots show the local covariance when starting from local minima ($\boldsymbol{s}^{\circ}$) at various Hamming distances from the global minimum ($\boldsymbol{s}^{\star}$). Error bars represent the standard deviation of bitstrings sampled at each d_{xy}. **a,** $\boldsymbol{s}^{\circ}$ with a Hamming distance of 10 from $\boldsymbol{s}^{\star}$. **b,** $\boldsymbol{s}^{\circ}$ with a Hamming distance of 20 from $\boldsymbol{s}^{\star}$. **c,** $\boldsymbol{s}^{\circ}$ with a Hamming distance of 30 from $\boldsymbol{s}^{\star}$.

C. Parameter setting scheme

QAOA is a variational algorithm that employs alternating cost and mixer layers, with γ_l (β_l) donate the parameter for the l-th cost (mixer) layer. Traditionally, these parameters are optimized through classical optimizer, with the loss function evaluated on a quantum processor. Recent work by Shree Hari Sureshbabu *et al.* [21] established near-optimal QAOA parameters for arbitrary weighted Ising model, by transferring parameters classically derived from infinite size unweighted Ising problems. We utilize these near-optimal parameters to construct our quantum sampler.

The procedure to construct a [L,Q]-sampler is as follows:

1. The QAOA parameters γ_l^{inf}, β_l^{inf} (for the l-th layer $l = 1, \ldots, Q$) optimized for large-girth regular unweighted graphs in the infinite-size limit are prepared in advance [12]. We then select the parameters of the first L layers from this set.

2. Given the Ising problem with J_{jk} and h_j, calculate the rescale factor A to account for weight distribution of the Ising model, as suggested in works [21]:

$$A = \sqrt{\frac{1}{C_J} \sum_{\{j,k\} \in N} J_{jk}^2 + \frac{1}{C_h} \sum_{j=1}^{N} h_j^2}, \tag{S5}$$

where C_J and C_h are the number of non-zero elements in J_{jk} and h_j.

3. Calculate the average degree of the graph D:

$$D = \frac{C_J}{N}. \tag{S6}$$

4. The final QAOA parameters are given by:

$$\gamma_l = A \arctan \frac{1}{\sqrt{D-1}} \times \gamma_l^{\text{inf}}, \quad \beta_l = \beta_l^{\text{inf}}. \tag{S7}$$

3. NUMERICAL SIMULATIONS

This section outlines the numerical simulation details for the experiment, including problem instance generation, the simulated annealing algorithm employed, and time analysis of the various components within Qjump.

A. Problem instances

All instances in this experiment are 2D Ising models with external fields, and their connectivity is shown in main text Fig. 2a. To align with the chip's topology, we used a rotated lattice, which maximizes the utilization of the qubits. The number of qubits in the lattice scales as $2 \times L \times (L+1)$ with increasing edge lengths. For better illustration, we selected instances with 60, 84, and 104 qubits, corresponding to $L = 5, 6$, and ~ 7. We focus on the following Ising model:

$$H_{\text{Ising}} = -\sum_{\{j,k\}\in N} J_{jk}\sigma_j^z\sigma_k^z - \sum_{j=1}^{N} h_j\sigma_j^z, \quad J_{jk} \sim \mathcal{N}(0,4), \quad h_j \sim \mathcal{N}(0,1), \tag{S8}$$

where qubits are arranged in a square lattice with only nearest-neighbor couplings, matching the 2D connectivity of our quantum processor.

In the field of optimization, the no-free-lunch theorem states that any two classical optimization algorithms should, on average, perform equally well across all possible optimization instances. To better study the performance of quantum optimization algorithms and their differences from classical algorithms, we filtered the benchmark instances. Given that practical optimization often involves running multiple algorithms in parallel and selecting the best result, our interest lies less in instances where classical algorithms like SA perform well. Instead, we focus on challenging instances where classical algorithms struggle and where quantum computing might offer an improvement.

The filtering process involved generating 4000 random Ising problems according to Eq. S8, solving them using a specialized SA solver (SimAn [22]), and selecting the 50 instances with the highest time to solution (TTS). To further filter out instances where classical jump algorithms perform well, we selected the top 20 instances with the most challenging classical jumps as our final benchmark instances. The corresponding coupling J_{jk} and local magnetic field h_j of problem instances for different system sizes are presented in Fig. S5, S6 and S7. The color of each circle indicates the magnitude of h_j, while the color of the connecting lines represents the magnitude of J_{jk}.

We excluded instances with fewer than 60 qubits for two primary reasons. First, the scaling curve is not apparent for smaller instances, only becoming stable and significant for larger instances. Second, smaller problem spaces are easier to solve without iterative methods, as the entire solution space can be explored by random initialization.

B. Simulated annealing

Simulated annealing (SA) is a powerful heuristic algorithm widely used for combinatorial optimization due to its simplicity and effectiveness. For the TTS estimation, we employed the SimAn software package [22], a highly optimized SA implementation executed on a single-core CPU (2.3 GHz) as a classical baseline. The effectiveness of SA is based on two facts, a high initial acceptance of any bit flips and a low escaping rate at later stages to prevent the solution from escaping the final minimum. For each problem instance, we randomly generated 10 bitstrings to calculate the average bit flip energy $\overline{|\Delta E|}$. The initial temperature T_0 is set to accept this $\overline{|\Delta E|}$ with 90% probability ($T_0 = -\overline{|\Delta E|}/\ln 0.90$), and the final temperature T_{end} is set to $0.01T_0$. The corresponding algorithmic workflow is outlined in Algorithm 1 and Fig. S8, and details referring to the procedure and code can be found elsewhere [22].

Notably, this implementation of SA is highly efficient for Ising problems, incorporating several key optimizations such as:

1. ΔE_j Forward computation: Precompute and store ΔE_j for each bit, with updates limited to cases where bit s_j or its neighbors are flipped.

2. Precomputation and reuse of random numbers across multiple annealing runs. To improve efficiency, the Metropolis criterion is modified from $\exp(-\Delta E_j/T) < u_j$ (where u_j is randomly chosen in $(0,1]$ and T is the annealing temperature of SA) to the equivalent $\Delta E_j < r_j$ (with $r = -T\log u_j$), allowing for precomputed values of r_j to be stored instead of u_j.

3. Optimization of loops via fixed lengths. The maximum number of neighbors is specified at compile time to enable more efficient compiler unrolling.

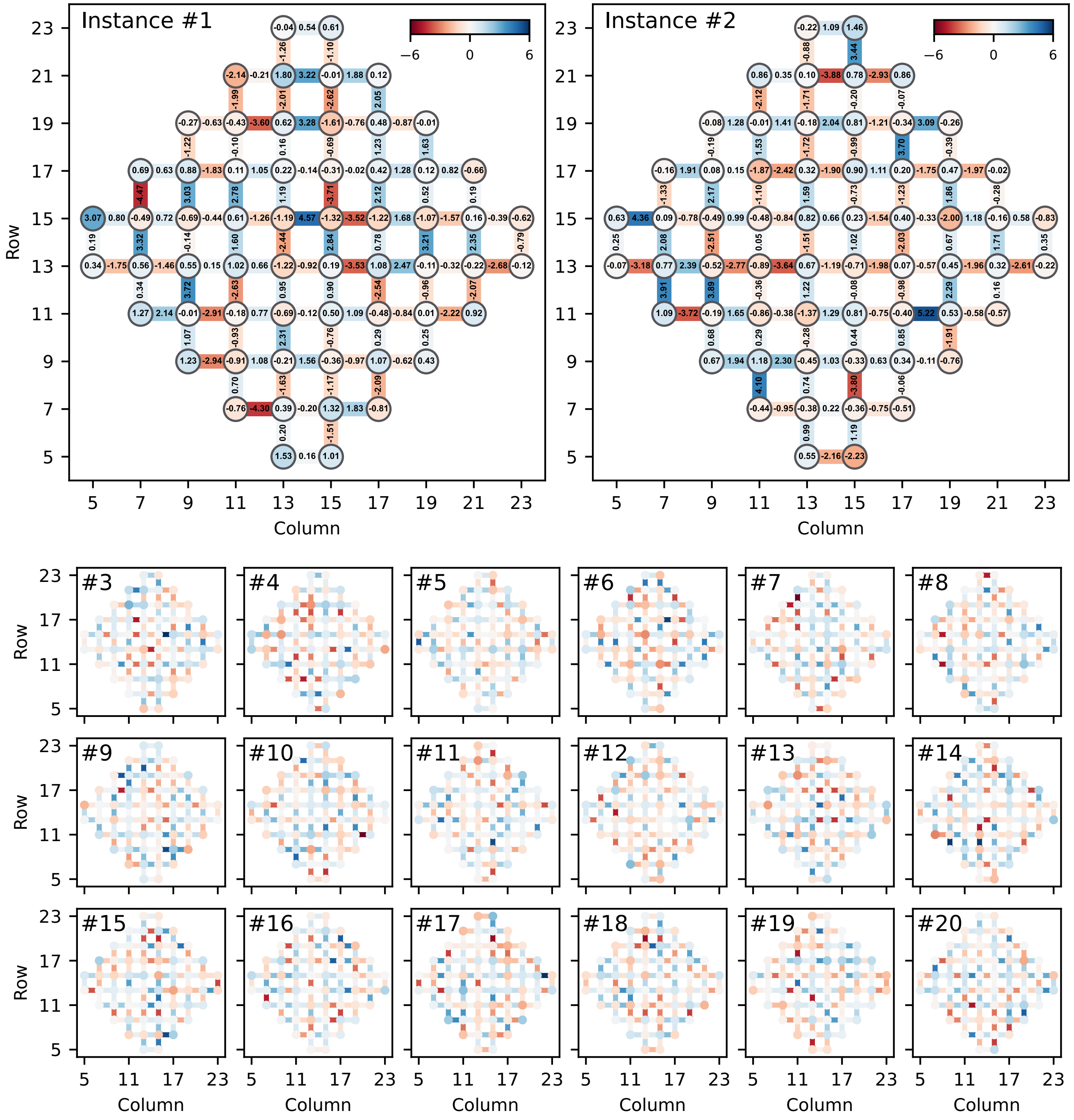

Figure S5. **Problem instances for** $N = 60$**.**

4. Deterministic lattice traversal. Lattice sites are updated in a predefined sequential order rather than being randomly selected.

5. Fast random number generators: Given that random number quality is less critical for optimization algorithms (unlike high-accuracy physical simulations), fast generators (e.g., linear-congruential) are employed to reduce computational overhead.

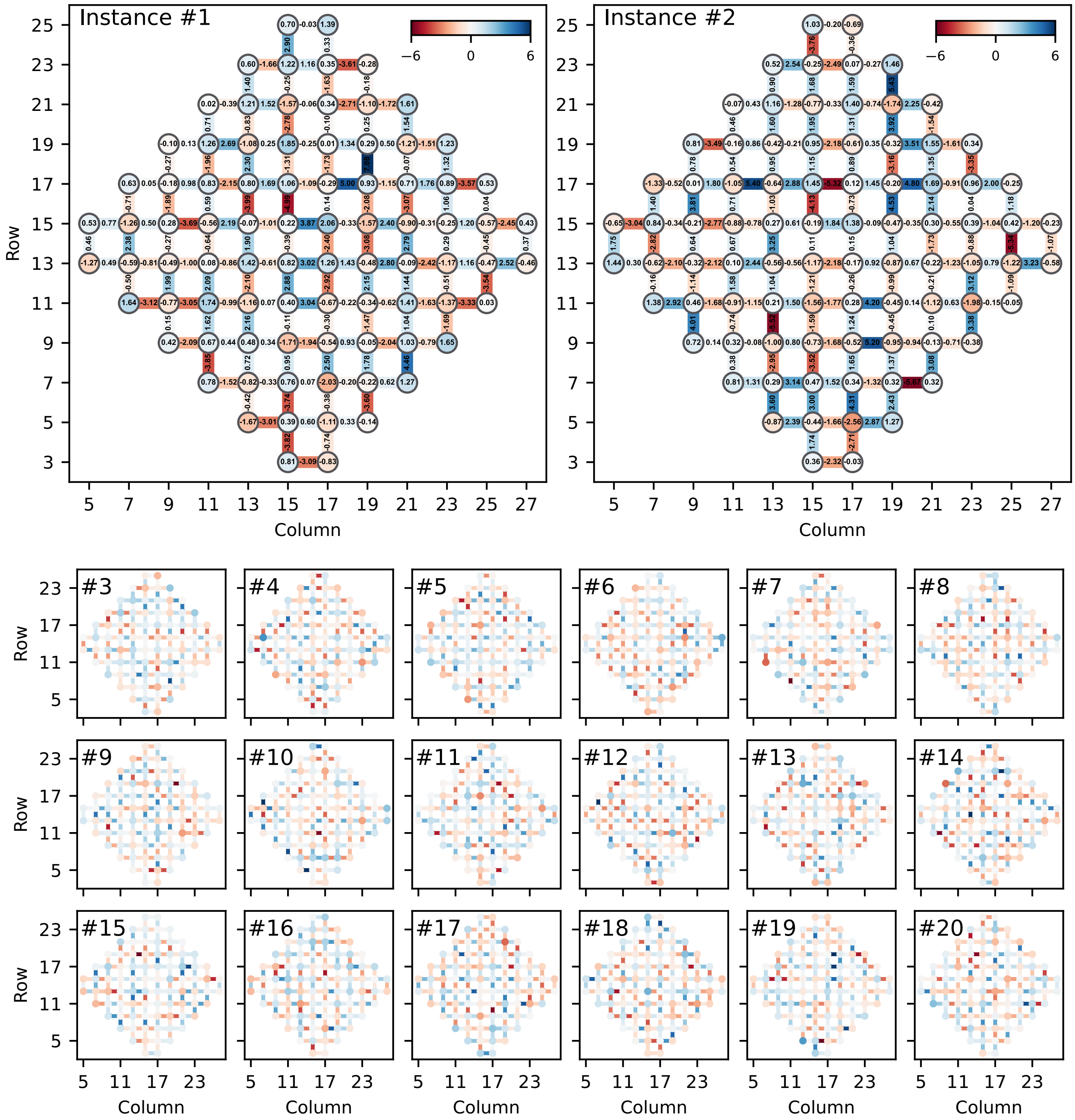

Figure S6. **Problem instances of $N = 84$.**

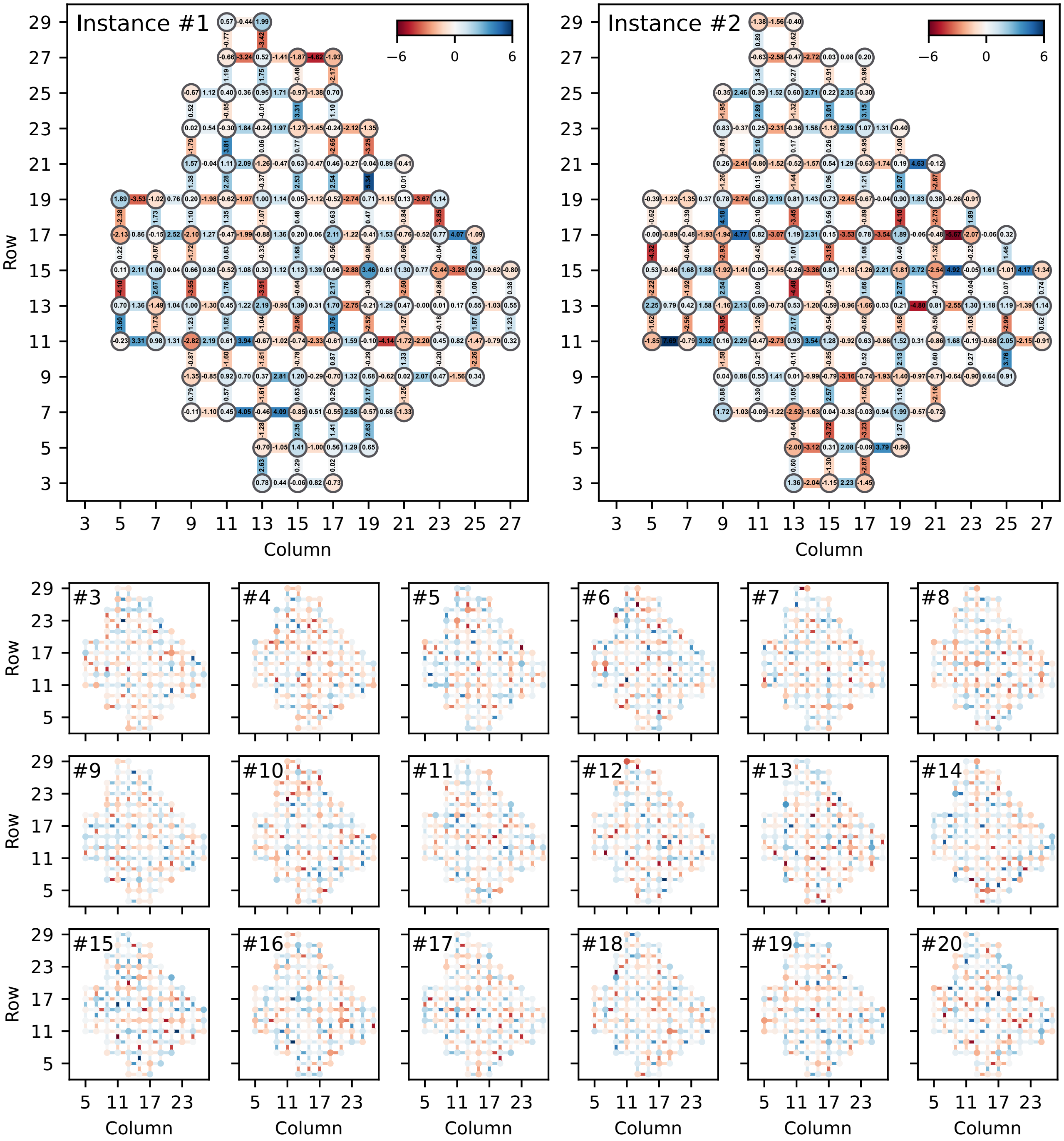

Figure S7. **Problem instances of** $N = 104$**.**

Algorithm 1 Simulated Annealing

Input: Ising model with couplings J_{jk} and fields h_j, initial temperature T_0, final temperature T_{end}, maximum sweeps $S_{\max}$
Output: Best solution found $\boldsymbol{s}^\star$
Generate initial guess solution $\boldsymbol{s}^\circ$
$\boldsymbol{s} \leftarrow \boldsymbol{s}^\circ$
$\boldsymbol{s}^\star \leftarrow \boldsymbol{s}^\circ$
$T \leftarrow T_0$
Precompute and store ΔE_j list for all bits $s_j \in \boldsymbol{s}^\circ$
For each T, precompute and store a list of random numbers $\{r_j\}$ corresponding to bits $\{s_j\}$, where $r_j = -T \ln u_j$ and u_j is randomly chosen in $(0, 1]$
for sweep = 1 to $S_{\max}$ **do**
    $T \leftarrow$ update according to schedule
    Shift the index of list $\{r_j\}$ at this temperature by a randomly generated integer
    for each bit s_j in fixed order **do**
        Retrieve stored random number r_j
        Retrieve stored ΔE_j
        if $\Delta E_j < r$ **then**
            Flip bit s_j in $\boldsymbol{s}$
            Update ΔE_k for $s_k \in \mathrm{neighbors}(s_j)$
            if the energy of $\boldsymbol{s}$ is lower than that of $\boldsymbol{s}^\star$ **then**
                $\boldsymbol{s}^\star \leftarrow \boldsymbol{s}$
            end if
        end if
    end for
end for
return $\boldsymbol{s}^\star$ with minimal energy

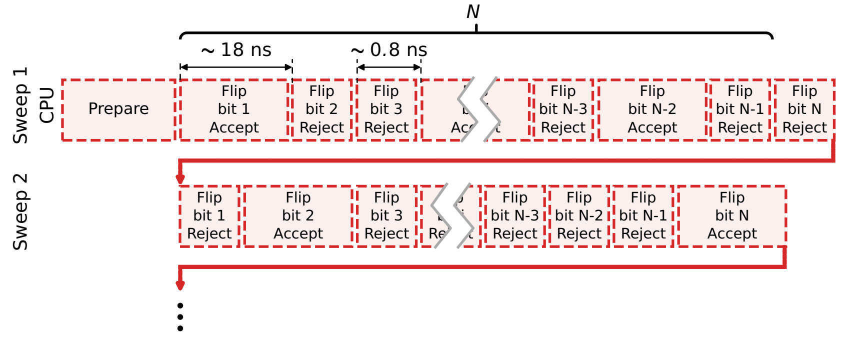

Figure S8. **Workflow of SA.** Preparation time is not included in SA runtime calculations, as this overhead does not scale with the number of runs. During each sweep, SA flips the bit one by one for all N bits, and each flip and corresponding operation may take 18 ns (accept) or 0.8 ns (reject) depending on whether the flipped bitstring is accepted by the Metropolis criterion.

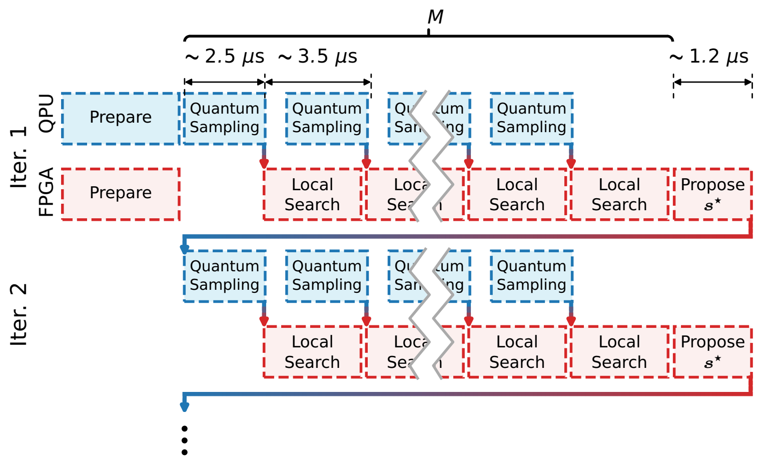

Figure S9. **Workflow of Qjump,** which is designed to run on an envisioned quantum hardware assuming that all control and data processing are on FPGA. The parallel programming ultimately minimizes the time cost of quantum operations for an optimal TTS. According to data reported in the literature, each quantum block (light blue) is speculated to take approximately 2.5 μs, which includes qubit reset (200 ns), qubit operations (33 layers and 40 ns per layer), qubit measurement (500 ns), and feedback control (500 ns) [23–25]. Each classical block (light red) is estimated to take approximately 3.5 μs, which consists of calculating the energy of the sampled bitstring (~ 500 ns) and performing roughly 18 steps of steepest descent (~ 160 ns per step). Preparation time is not included in Qjump runtime calculations, as this overhead does not scale with the number of iterations or runs.

C. Qjump time estimation

As our current experimental setup lacks the essential elements for prompt and repeated execution of Qjump, such as rapid qubit reset, fast readout and feedback control, and in-situ data processing for local search, our analysis of the Qjump runtime can only be based on an envisioned Qjump hardware, with knowledge from the literature. The proposed hardware, with all operations instructed by an FPGA, is able to perform a sequence of operations including qubit reset, gate operation, measurement, classical local search and comparison, and feedback control to implement gate set for the next iteration. For quibt operations, we estimate the time scales by quoting data from the literature (qubit reset time 200 ns, gate time 40 ns, qubit measurement time 500 ns, and feedback control time 500 ns) [23–25]; for classical processing, since FPGA can be as fast as CPU, we simply use the time data recorded on CPU to estimate the time consumption of the classical local search on FPGA.

An optimized Qjump workflow is illustrated in Fig. S9, where the quantum sampling part (rows with blue blocks) starts one step ahead but runs almost in parallel with the classical local search part (rows with pink blocks) within $M = 20$ rounds. According to this workflow, the runtime for the Qjump algorithm with $L = 2$, $M = 20$, and 12 iterations is estimated to be ~0.78 ms , as mostly decided by the classical operations. In comparison, the runtime for the QAOA algorithm with $Q = 6$ is ~5.1 μs and that for the SA algorithm with 700 sweeps is estimated to be ~0.30 ms, respectively. In our current experimental setup, however, the wall-clock time for Qjump algorithm is approximately 2 s per iteration. This huge latency is dominated by the classical-quantum interface overhead, i.e., we use a personal computer to perform classical local search which communicates by Ethernet with the FPGA-based control and measurement electronics. In the envisioned quantum hardware, the local search role can be integrated into the FPGA of the control and measurement electronics, significantly suppressing the latency time[23].

The overall Qjump runtime, as shown in Fig. S9, is primarily constrained by its classical computation time. This classical computation mainly involves two steps: performing a local search on each sampled result (with a time of t_{LS}) and proposing the optimal result from those retained in each iteration (with a time of t_{PO}). Specifically, the local search, shown in Algorithm 2, decomposes into calculating the total energy of the sampled bitstring and applying steepest descent to find the local minima. These steps further break down into two fundamental operations: bit flipping and updating the energy list (with a time of t_{SF}), and identifying the minimum within the energy list (with a time of t_{ML}), which are highlighted with yellow and pink, respectively. The runtime of local search for a candidate solution $\boldsymbol{s}$ can be formalized as:

$$t_{\mathrm{LS}}(N, n_{\mathrm{LS}}) = \eta \times N \times t_{\mathrm{SF}} + n_{\mathrm{LS}} \times (t_{\mathrm{ML}} + t_{\mathrm{SF}}),$$

Here, N is the system size and n_{LS} is the number of descent steps. η is the average bit flip ratio for the quantum

Algorithm 2 Greedy Local Search

Input: Ising model with couplings J_{jk} and fields h_j, system size N, initial guess of this iteration $\boldsymbol{s}^{\circ}$, energy of the initial guess E°, candidate solution $\boldsymbol{s}$, a list $\{\Delta E_j^{\circ}\}$, inherited from the previous Qjump iteration, recording the Ising energy difference ΔE_j before and after flipping $s_j \in \boldsymbol{s}^{\circ}$
Output: the local minima $\boldsymbol{s}^{\star}$, energy of the local minima $E^{\star}$, a list $\{\Delta E_j^{\star}\}$, recording the Ising energy difference before and after flipping $s_j \in \boldsymbol{s}^{\star}$
$j \leftarrow 1$
$E \leftarrow E^{\circ}$
$\{\Delta E_j\} \leftarrow \{\Delta E_j^{\circ}\}$
$n_{\text{LS}} \leftarrow 0$
/* Calculate the energy of the candidate solution $\boldsymbol{s}$ */
for $j \leq N$ **do**
    $s_j^{\circ} \in \boldsymbol{s}^{\circ}$
    $s_j \in \boldsymbol{s}$
    if $s_j \neq s_j^{\circ}$ **then**
        $E \leftarrow E + \Delta E_j$
        Update ΔE_j, $\Delta E_k \in \{\Delta E_j\}$ for $s_k \in$ neighbors(s_j)
    end if
end for

/* Applying steepest descent to $\boldsymbol{s}$ */
while True **do**
    Find $j^{\star}$ where $\Delta E_{j^{\star}}$ is minimized in $\{\Delta E_j\}$
    if $\Delta E_{j^{\star}} < 0$ **then**
        Flip bit $s_{j^{\star}}$ in $\boldsymbol{s}$
        $E \leftarrow E + \Delta E_{j^{\star}}$
        Update $\Delta E_{j^{\star}}$, $\Delta E_k \in \{\Delta E_j\}$ for $s_k \in$ neighbors$(s_{j^{\star}})$
        $n_{\text{LS}} \leftarrow n_{\text{LS}} + 1$
    else
        break
    end if
end while

$\boldsymbol{s}^{\star} \leftarrow \boldsymbol{s}$
$E^{\star} \leftarrow E$
$\{\Delta E_j^{\star}\} \leftarrow \{\Delta E_j\}$
return $\boldsymbol{s}^{\star}$, $E^{\star}$, $\{\Delta E_j^{\star}\}$

sampler. The first term represents the energy calculation time, while the second term denotes the steepest descent time.

To benchmark these individual time components (t_{SF}, t_{ML} and t_{PO}), we executed the classical portion of Qjump on the same classical platform used for SA, with the quantum sampler replaced by a random bit flip mechanism. We performed 100 runs each on all 20 instances selected in Section 3A, each consisting of 20 iterations, and recorded the time consumption for each component. Notably, t_{PO} also includes overheads for maintaining variables, copying data, and memory management within each iteration, along with an additional 200 ns delay representing the FPGA-to-QPU solution transfer time. The average classical time costs for the system structure presented in the main text and the additional experimental data in Section 4D are summarized in Table S1.

System size N	60	84	104
t_{SF} (ns)	25.3	27.8	28.6
t_{ML} (ns)	87.7	107.6	128.2
t_{PO} (ns)	1378.8	1260.6	1249.2

Table S1. Time consumption for individual components in the classical portion of Qjump.

For a rough approximation, we also estimated the base classical operation costs from CPU specifications. Given the single-core CPU frequency of 2.3 GHz, one clock cycle is approximately 0.43 ns. For a system with connectivity $D \sim 4$, bit flipping and updating the energy list (t_{SF}) involves roughly 50 clock cycles, leading to t_{SF} ~20 ns. The time to identify the minimum within the energy list (t_{ML}) is similarly estimated to be ~100 ns (at $N = 104$), based on a brute-force search requiring $\sim 2N$ clock cycles. We used this brute-force approach because more complex sorting

algorithms offered no significant advantage for $N \sim 100$ due to the overhead of maintaining additional data structures. Both values are consistent with the runtime recorded.

To further analyze the time complexity of the classical portion, we extended our numerical algorithm to larger system sizes and higher degrees. We generated one random instance for each system size (N) ranging from 100 to 600 while keeping the degree fixed at 4. To investigate the scaling with respect to connectivity, we also generated one random instance for $N = 600$ at various degrees (D) from $0.2N$ to $0.8N$. The time consumption for each component of the numerical algorithm on these instances is presented in Fig. S10.

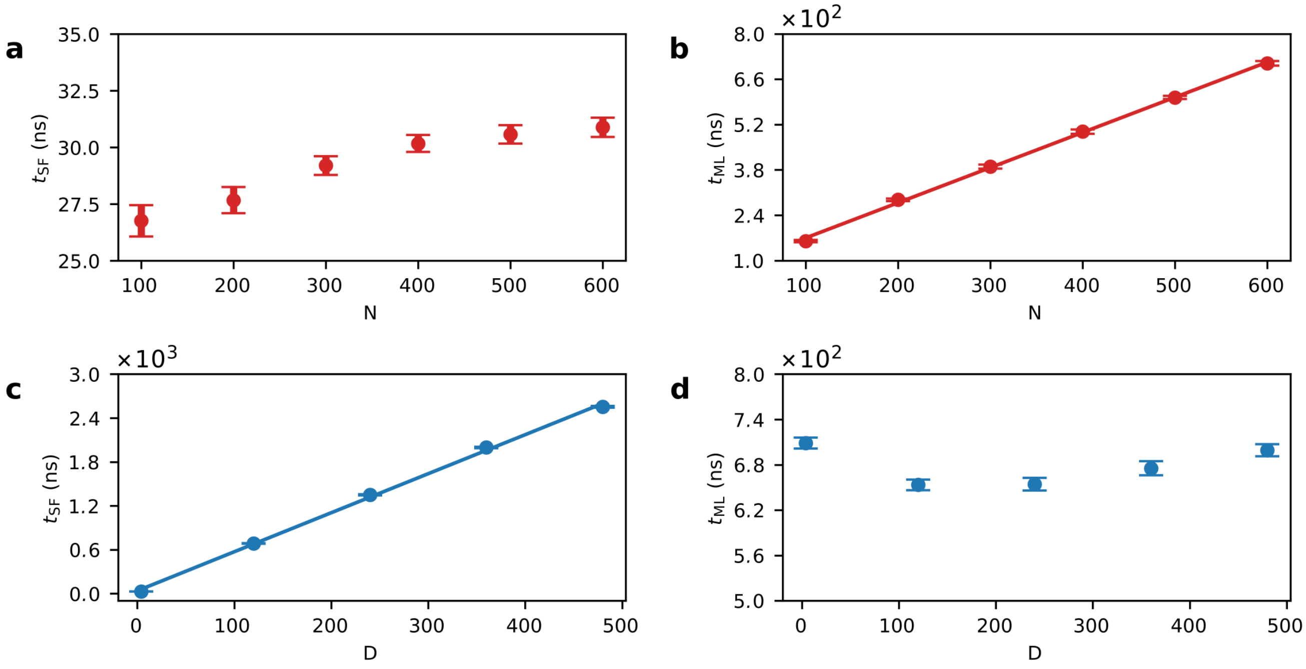

Figure S10. **Runtime benchmark and scaling behavior of individual time components in the classical portion of Qjump.** The data points represent the mean time consumption, while the error bars indicate the standard deviation over 100 runs. The solid lines show the linear fit to the data.

As shown in Fig. S10**a** and **c**, t_{SF} is primarily determined by graph connectivity D, rather than the system size N, due to an incremental gradient maintenance method yielding $O(D)$ time complexity. For smaller system sizes, boundary effects reduce connectivity at edge qubits, leading to a smaller and more volatile t_{SF}. Conversely, t_{ML} scales as $O(N)$ because it requires a traversal of all bits to find the largest improvement. Our numerical data corroborate this behavior, as shown in Fig. S10**b** and **d**.

4. EXPERIMENTAL DETAILS

In this section, we present the key parameters and performance metrics of our 104-qubit quantum processor (Sec. 4A). We provide a detailed description of the quantum circuits implemented in the Qjump algorithm (Sec. 4B), along with trials for different parameter settings used in Qjump (Sec. 4C). Finally, we provide additional experimental data for different qubit numbers (Sec. 4D).

A. Device infomation

The wiring information and room temperature control electronics are similar to those of [26]. We summarize single-qubit parameters including idle frequency, readout error averaged for qubit in $|0\rangle$ and $|1\rangle$, energy relaxation time T_1, and Hahn echo dephasing time T_2^{SE} in Fig. S11. The readout error was measured by initializing all qubits into random product states. To improve measurement fidelity, an additional microwave pulse inducing the $|1\rangle \leftrightarrow |2\rangle$ transition is applied to each qubit before the readout.

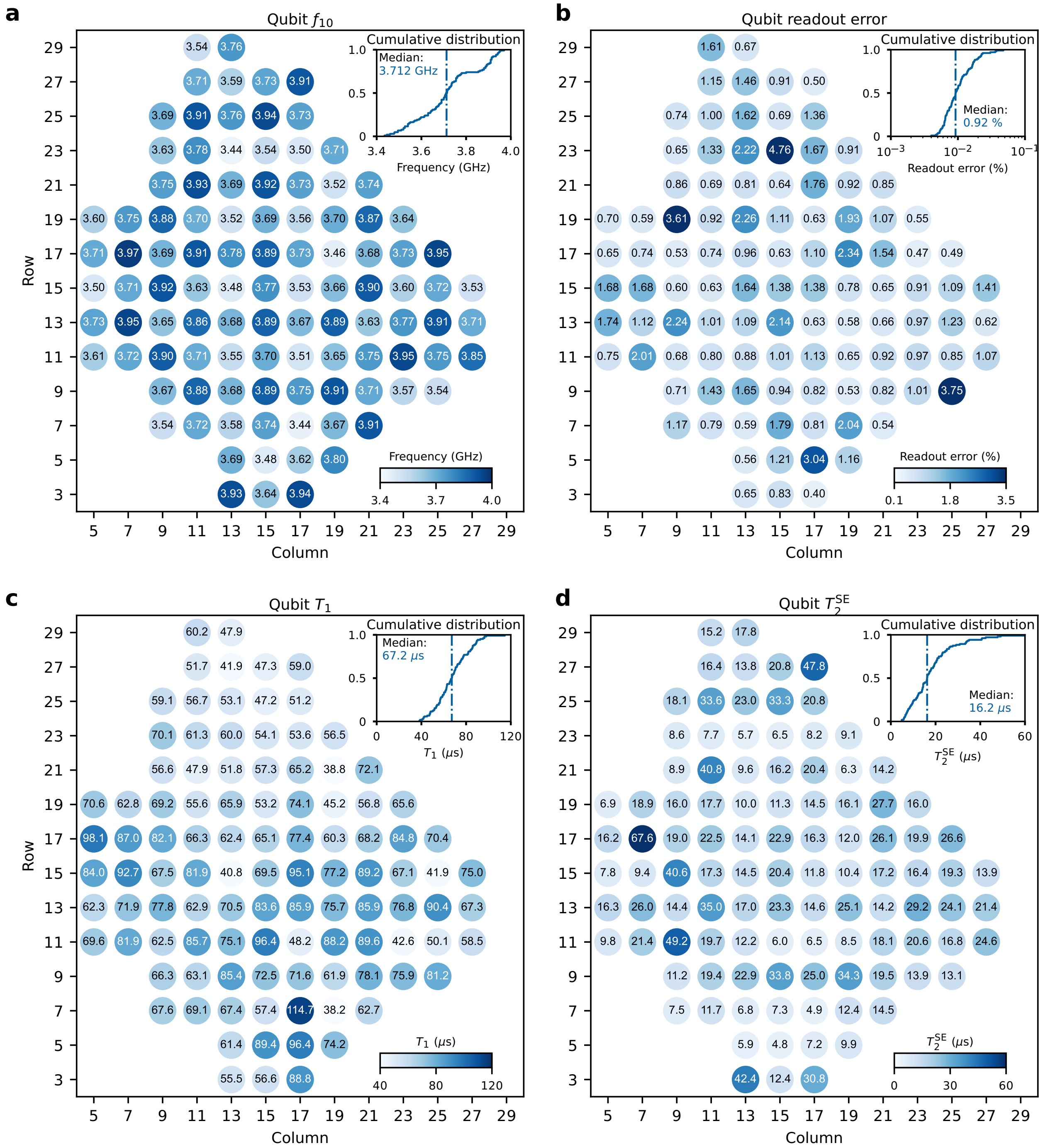

Figure S11. **Heat map of single-qubit parameters.** **a,** Qubit idle frequency. **b,** Readout error averaged for qubit in $|0\rangle$ and $|1\rangle$, measured by preparing random product states on all qubits. The median for 104 qubits is 0.92%. **c,** Qubit relaxation time measured at its idle frequency. The median for 104 qubits is 67.2 μs. **d,** Qubit dephasing time measured using Hahn echo sequence at its idle frequency. The median for 104 qubits is 16.2 μs.

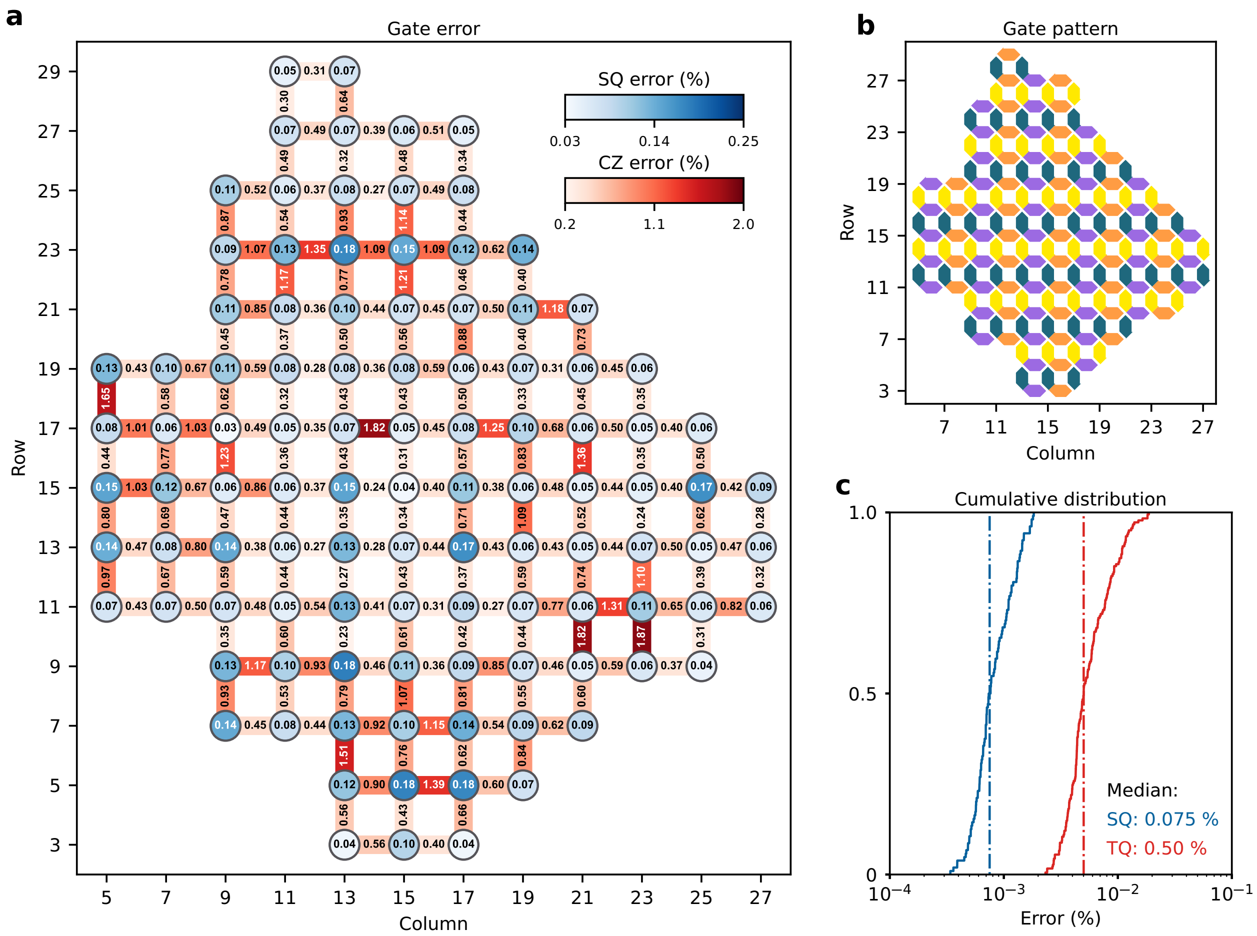

Figure S12. **Pauli errors of single- and two-qubit gates.** **a,** Heatmap of Pauli errors. Gate errors are benchmarked by simultaneous XEB. Two-qubit CZ gate errors are measured based on gate patterns shown in **b**. **b,** Two-qubit gate patterns indexed by four different colors of the corresponding couplers. Gates with the same coupler color are benchmarked simultaneously. **c,** Cumulative distribution of Pauli errors of single- and two-qubit gates. Dashed lines indicate the median values.

Single-qubit gates XY rotations are realized using microwave pulses with 20 ns duration. Two-qubit diabatic controlled-Z (CZ) gate is implemented by tuning the $|11\rangle$ and $|20\rangle$ energy levels of a qubit pair to near resonance and meanwhile activating the coupling between qubits. Figure S12b illustrates how the tunable couplers are divided into four subsets, with all corresponding CZ gates in the same subset running simultaneously. We benchmark the performance of single- and two-qubit gates by simultaneous cross-entropy benchmarking (XEB), yielding median fidelities of single- and two-qubit gates around 99.95% and 99.5%, respectively (Fig. S12).

B. Quantum circuit

The experiment circuit, as depicted in Fig. 1**b** and Fig. 2**b** of the main text, is further compiled for hardware execution. This compiling process aims to optimize performance and mitigate errors, involving the following steps:

1. Pauli twirling: To mitigate coherent error accumulation, native CZ gates are implemented with equivalent gates that incorporate two layers of single-qubit rotations on both sides [27, 28].

2. Merging sequential single-qubit gates: Consecutive single-qubit gates are merged into a microwave XY rotation combined with a virtual Z rotation, thereby reducing the total number of single-qubit gates.

3. Gate arrangement: Gates are arranged in the sequence to avoid the case of running single- and two-qubit gates in parallel.

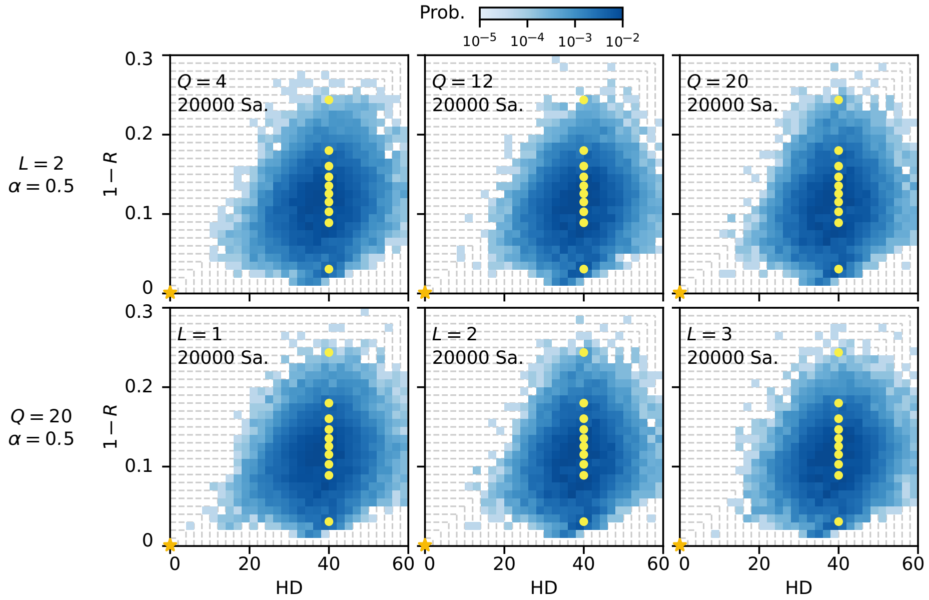

Figure S13. **Performance of the quantum sampler with different L and Q values.** Top-row panels display the sampling probability after local search ($P^\star$) for the quantum sampler, with varying Q values at $L = 2$. Bottom-row panels present $P^\star$ with varying L values at $Q = 20$. All cases start with 10 initial $\boldsymbol{s}^\circ$ (highlighted by yellow circles) at the Hamming distance of 40 from the global optimal bitstring (highlighted by golden star). The background gray dashed lines outline a series of square regions that approach the global minimum, where increasing proximity to the bottom-left corner indicates a closer distance to the optimum.

4. Dephasing suppression: Two X-gates (π rotations around X-axis in the Bloch sphere) are applied to qubits that idle more than 150 ns.

C. Performance of the quantum sampler with different parameters

We experimentally tested various parameters of the quantum sampler, including Q up to 20, L from 1 to 3 and $\alpha \in [0.3, 0.7]$ for Ising instance #1 at $N = 104$, with all configurations demonstrating similar behaviors. The sampling performance for different values of L and Q with a fixed $\alpha = 0.5$ is presented in Fig. S13. The performance of the [2, 20]-sampler with $\alpha \in [0.3, 0.7]$ is shown in Fig. S14, along with comparison to classical random sampling with similar bit flip ratios.

D. Additional experimental data for different system sizes

In this section, we provide supplementary figures for Ising problems with $N = 60$ and $N = 84$. The qubit layouts for both systems are shown in Fig. S15 and Fig. S18, along with the corresponding performance of single-qubit gates, CZ gates, and readout. Comparisons of Qjump's performance against SA and QAOA for these same system sizes are shown in Fig. S16 and Fig. S19. The Qjump iteration number and the SA sweep number are optimized for each system size based on the TTS metric, and the corresponding TTS values are shown in Fig. S17 and Fig. S20. Due to the interface-induced latency in our current quantum hardware, for each instance, the TTS for Qjump is estimated from 50 independent runs. In contrast, 10^5 repetitions are employed for SA to ensure a high-precision baseline for comparison.

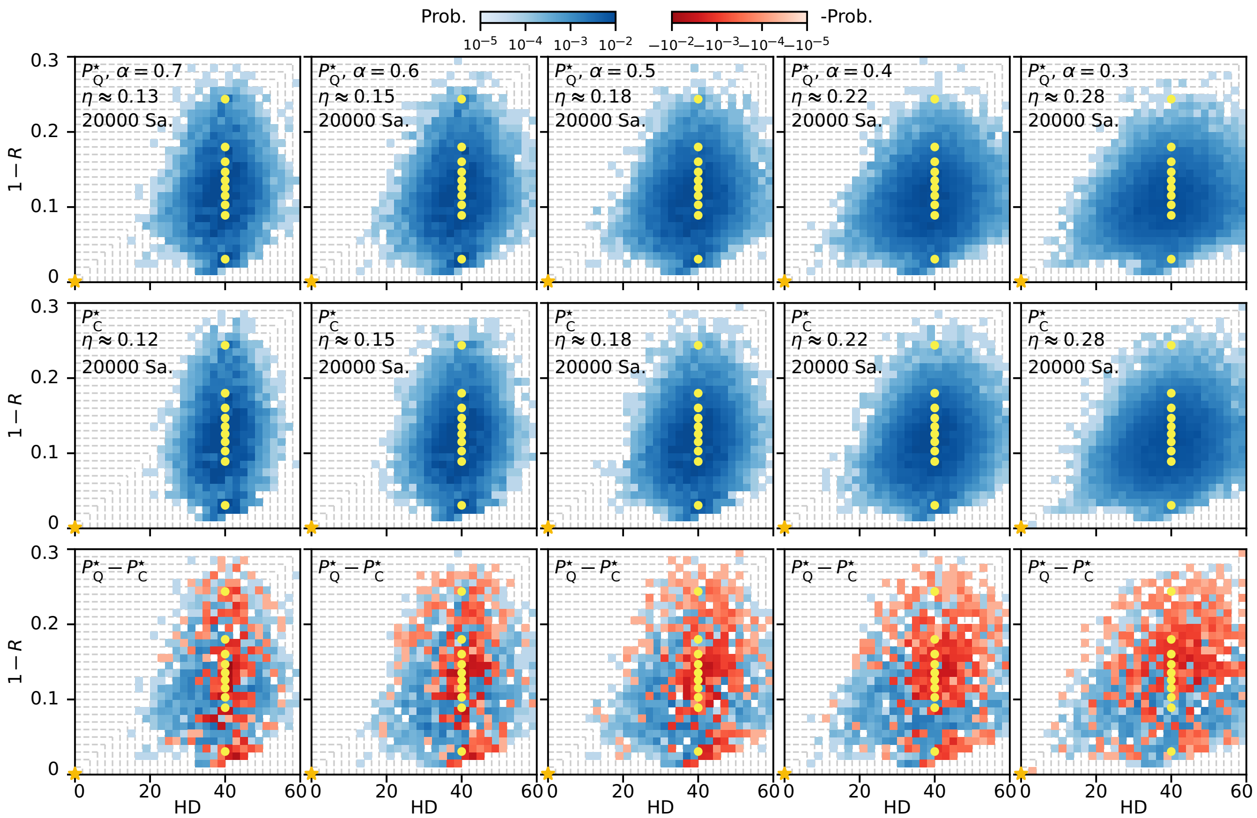

Figure S14. **Performance of the quantum sampler with different α values.** Top-row panels display the sampling probability after local search ($P^\star_Q$) for the quantum sampler, with varying α at $L = 2$ and $Q = 20$. Equivalent bit flip ratios η are labeled. Middle-row panels present $P^\star_C$ by classical random sampling with comparable η values. Bottom-row panels show the difference between $P^\star_Q$ and $P^\star_C$.

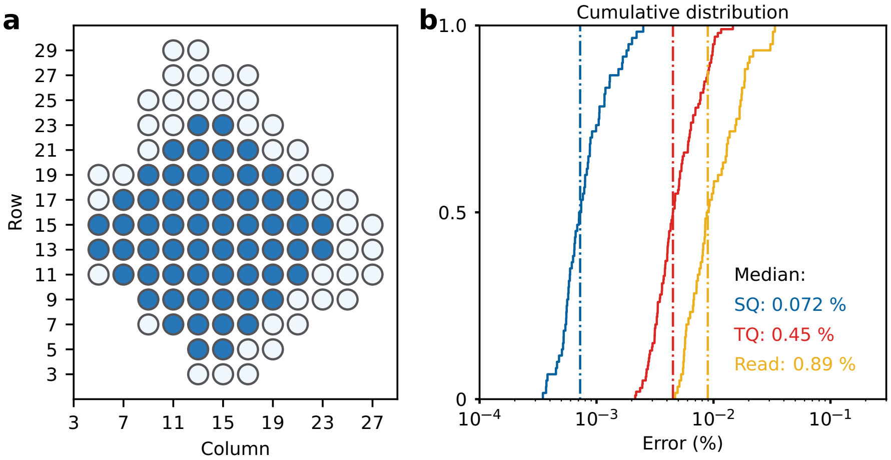

Figure S15. **Device information for $N = 60$ experiment.** **a,** 2D lattice of qubits selected for $N = 60$ experiment. **b,** Cumulative distribution of Pauli errors for single- and two-qubit gates, alongside the distribution of readout errors for $N = 60$. Dashed lines indicate the median values.

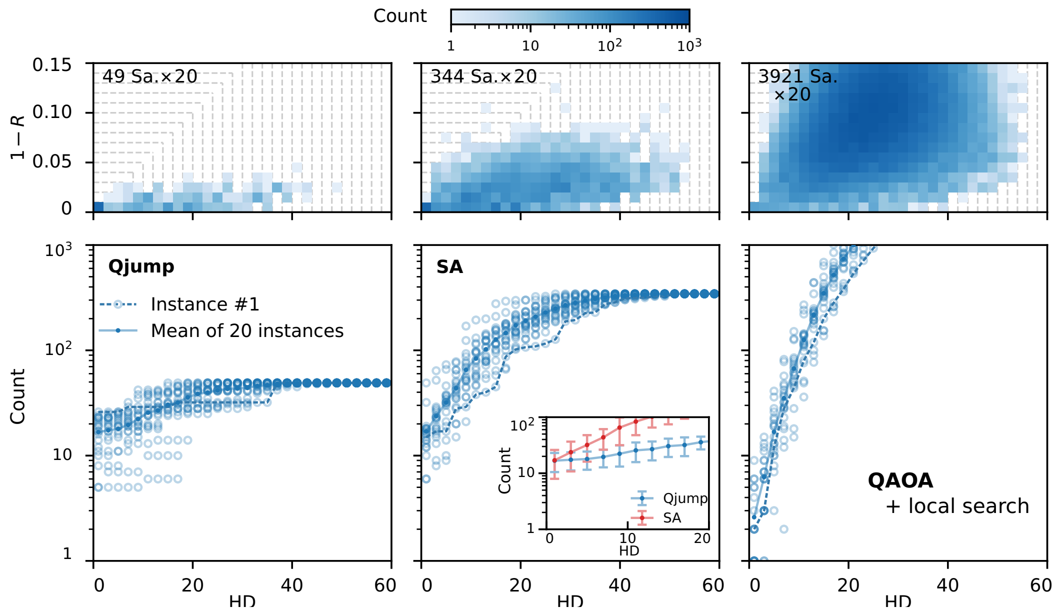

Figure S16. **Performace of Qjump, QAOA and SA for** $N = 60$**.** This figure summarizes the solution distributions of Qjump, SA, and QAOA across 20 problem instances. Top row: solution count distribution over 20 problem instances for each algorithm. Bottom row: solution count for individual instances and the means as functions of HD toward the global minimum. For this comparison, we execute Qjump with 8 iterations at $\alpha = 0.5$, SA with 200 sweeps, and QAOA with $Q = 6$ and extract the lowest-energy bitstring from each run for analysis. Based on the computational speed of the envisioned quantum hardware and classical CPU (time per run: Qjump ~ 0.41 ms, SA ~ 0.06 ms, QAOA ~ 5.1 μs), we first determine the numbers of bitstrings that can be produced by these algorithms in repeated runs within a fixed period of 20 ms, and then run Qjump and QAOA on our superconducting processor to generate the pre-determined numbers of bitstrings for analysis, which takes a much longer time on our experimental setup. For this small system size, the runtime of Qjump is primarily limited by the quantum sampling frequency rather than the classical local search. Mean values extracted from Qjump and SA are shown in the panel inset, with error bars representing the standard deviations over 20 instances. On average, Qjump samples the global minimum 0.98 times as frequently as SA.

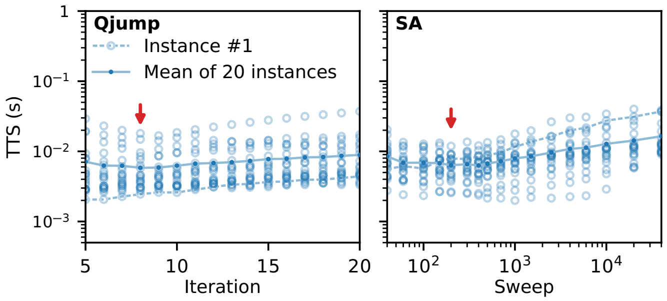

Figure S17. **TTS estimation for Qjump and SA at** $N = 60$**.** Each empty circle represents the TTS value for a certain problem instance obtained by running the Qjump (SA) algorithm with the specified iteration (sweep) number. Dots connected by lines indicate the mean of the data for the 20 problem instances, and the arrow points to the optimal iteration/sweep number used for the algorithmic benchmark in Fig. S16. TTS values for instance #1 are highlighted by dashed lines. According to the minimal TTS values, on average, Qjump running on the envisioned quantum hardware demonstrates comparable performance to SA, with a TTS ratio of approximately 1.09.

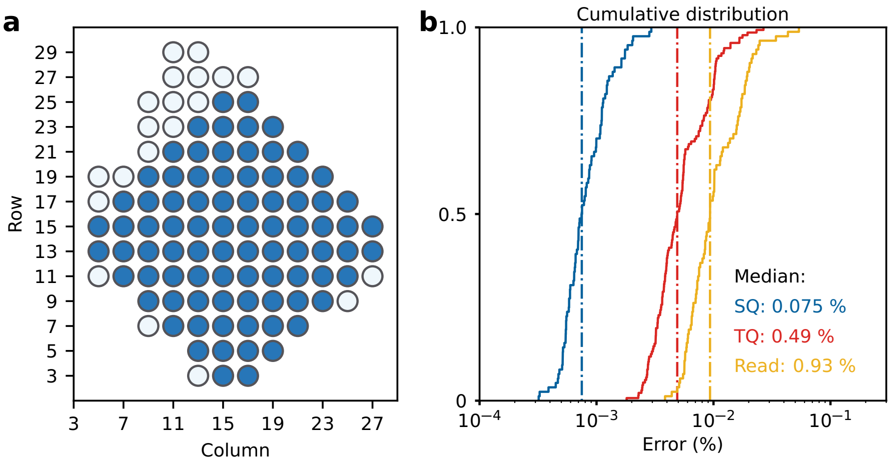

Figure S18. **Device information for $N = 84$ experiment.** **a,** 2D lattice of qubits selected for $N = 84$ experiment. **b,** Cumulative distribution of Pauli errors for single- and two-qubit gates, alongside the distribution of readout errors for $N = 84$. Dashed lines indicate the median values.

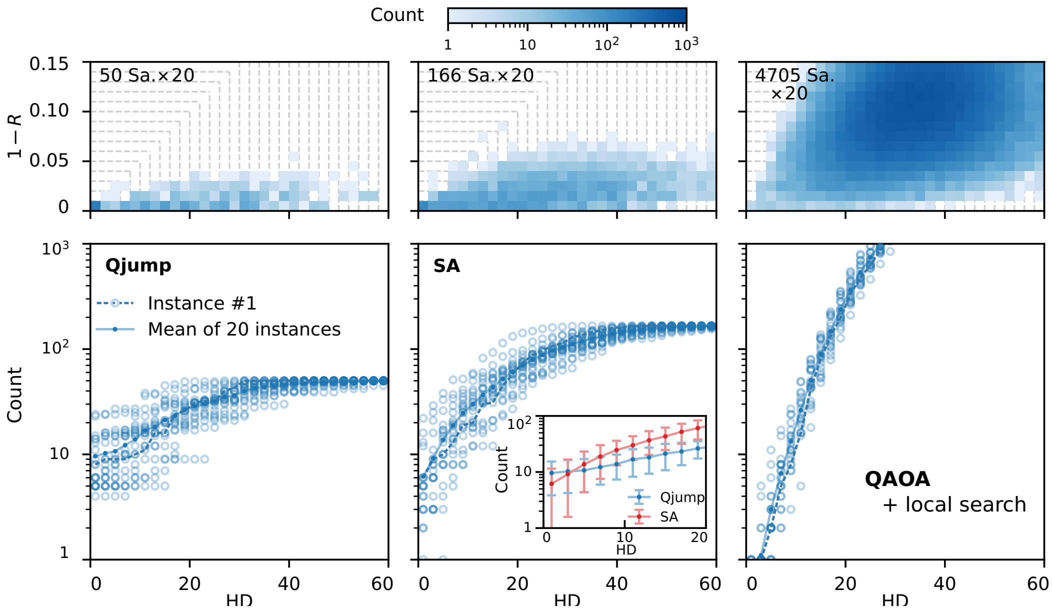

Figure S19. **Performace of Qjump, QAOA and SA for $N = 84$.** This figure summarizes the solution distributions of Qjump, SA, and QAOA across 20 problem instances. Top row: solution count distribution over 20 problem instances for each algorithm. Bottom row: solution count for individual instances and the means as functions of HD toward the global minimum. For this comparison, we execute Qjump with 9 iterations at $\alpha = 0.5$, SA with 400 sweeps, and QAOA with $Q = 6$ and extract the lowest-energy bitstring from each run for analysis. Based on the computational speed of the envisioned quantum hardware and classical CPU (time per run: Qjump ~ 0.47 ms, SA ~ 0.14 ms, QAOA ~ 5.1 μs), we first determine the numbers of bitstrings that can be produced by these algorithms in repeated runs within a fixed period of 24 ms, and then run Qjump and QAOA on our superconducting processor to generate the pre-determined numbers of bitstrings for analysis, which takes a much longer time on our experimental setup. Mean values extracted from Qjump and SA are shown in the panel inset, with error bars representing the standard deviations over 20 instances. On average, Qjump samples the global minimum 1.56 times more frequently than SA.

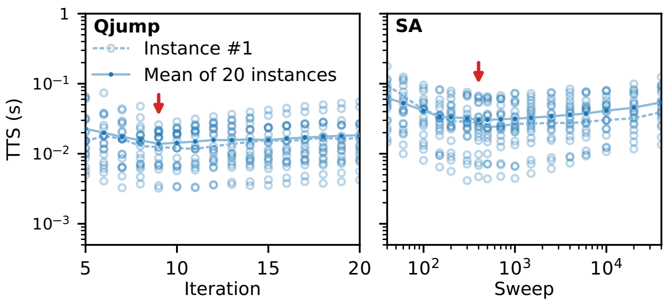

Figure S20. **TTS estimation for Qjump and SA at** $N = 84$**.** Each empty circle represents the TTS value for a certain problem instance obtained by running the Qjump (SA) algorithm with the specified iteration (sweep) number. Dots connected by lines indicate the mean of the data for the 20 problem instances, and the arrow points to the optimal iteration/sweep number used for the algorithmic benchmark in Fig. S19. TTS values for instance #1 are highlighted by dashed lines. According to the minimal TTS values, on average, Qjump running on the envisioned quantum hardware outperforms SA by roughly a factor of 2.18.

[1] Kirkpatrick, S., Gelatt, C. D. & Vecchi, M. P. Optimization by simulated annealing. *Science* **220**, 671–680 (1983).
[2] Glover, F. Tabu search—part i. *ORSA J. Comput.* **1**, 190–206 (1989).
[3] Holland, J. H. Genetic algorithms. *Sci. Am.* **267**, 66–73 (1992).
[4] Lucas, A. Ising formulations of many NP problems. *Front. Phys.* **2**, 5 (2014).
[5] Farhi, E., Goldstone, J. & Gutmann, S. A quantum approximate optimization algorithm. *arXiv:1411.4028* (2014).
[6] Blekos, K. *et al.* A review on quantum approximate optimization algorithm and its variants. *Phys. Rep.* **1068**, 1–66 (2024).
[7] McArdle, S. *et al.* Variational ansatz-based quantum simulation of imaginary time evolution. *npj Quantum Inf.* **5**, 75 (2019).
[8] Wang, X. *et al.* Imaginary Hamiltonian variational ansatz for combinatorial optimization problems. *Phys. Rev. A* **111**, 032612 (2025).
[9] Marsh, S. & Wang, J. B. Combinatorial optimization via highly efficient quantum walks. *Phys. Rev. Res.* **2**, 023302 (2020).
[10] Kadowaki, T. & Nishimori, H. Quantum annealing in the transverse Ising model. *Phys. Rev. E* **58**, 5355 (1998).
[11] Munoz-Bauza, H. & Lidar, D. Scaling advantage in approximate optimization with quantum annealing. *Phys. Rev. Lett.* **134**, 160601 (2025).
[12] Basso, J., Farhi, E., Marwaha, K., Villalonga, B. & Zhou, L. The quantum approximate optimization algorithm at high depth for MaxCut on large-girth regular graphs and the Sherrington-Kirkpatrick model. In *17th Conference on the Theory of Quantum Computation, Communication and Cryptography* (2022).
[13] Montanez-Barrera, J. A. & Michielsen, K. Towards a universal QAOA protocol: Evidence of a scaling advantage in solving some combinatorial optimization problems. *arXiv:2405.09169* (2024).
[14] Pelofske, E., Bärtschi, A. & Eidenbenz, S. Short-depth QAOA circuits and quantum annealing on higher-order Ising models. *npj Quantum Inf.* **10**, 30 (2024).
[15] Harrigan, M. P. *et al.* Quantum approximate optimization of non-planar graph problems on a planar superconducting processor. *Nat. Phys.* **17**, 332–336 (2021).
[16] Egger, D. J., Mareček, J. & Woerner, S. Warm-starting quantum optimization. *Quantum* **5**, 479 (2021).
[17] Tate, R., Moondra, J., Gard, B., Mohler, G. & Gupta, S. Warm-started QAOA with custom mixers provably converges and computationally beats Goemans-Williamson's Maxcut at low circuit depths. *Quantum* **7**, 1121 (2023).
[18] Díez-Valle, P., Porras, D. & García-Ripoll, J. J. Connection between single-layer quantum approximate optimization algorithm interferometry and thermal distribution sampling. *Front. Quantum Science and Technology* **3**, 1321264 (2024).
[19] Xu, X. *et al.* Mindspore quantum: A user-friendly, high-performance, and ai-compatible quantum computing framework. *arXiv:2406.17248* (2024).
[20] Díez-Valle, P., Porras, D. & García-Ripoll, J. J. Quantum approximate optimization algorithm Pseudo-Boltzmann states. *Phys. Rev. Lett.* **130**, 050601 (2023).
[21] Sureshbabu, S. H. *et al.* Parameter setting in quantum approximate optimization of weighted problems. *Quantum* **8**, 1231 (2024).
[22] Isakov, S. V., Zintchenko, I. N., Rønnow, T. F. & Troyer, M. Optimised simulated annealing for Ising spin glasses. *Comput. Phys. Commun.* **192**, 265–271 (2015).
[23] Salathé, Y. *et al.* Low-latency digital signal processing for feedback and feedforward in quantum computing and communication. *Phys. Rev. Appl.* **9**, 034011 (2018).
[24] Acharya, R. *et al.* Suppressing quantum errors by scaling a surface code logical qubit. *Nature* **614**, 676–681 (2023).
[25] Acharya, R. *et al.* Quantum error correction below the surface code threshold. *Nature* **638**, 920 (2024).
[26] Jin, F. *et al.* Topological prethermal strong zero modes on superconducting processors. *Nature* **645**, 626–632 (2025).
[27] Layden, D. *et al.* Quantum-enhanced Markov chain Monte Carlo. *Nature* **619**, 282–287 (2023).
[28] Wold, K. *et al.* Experimental detection of dissipative quantum chaos. *arXiv:2506.04325* (2025).
